# Impact of diabetes mellitus on tuberculosis prevention, diagnosis, and treatment from an immunologic perspective

**DOI:** 10.1002/EXP.20230138

**Published:** 2024-03-05

**Authors:** Zhaoyang Ye, Linsheng Li, Ling Yang, Li Zhuang, Ashok Aspatwar, Liang Wang, Wenping Gong

**Affiliations:** ^1^ Beijing Key Laboratory of New Techniques of Tuberculosis Diagnosis and Treatment Senior Department of Tuberculosis The Eighth Medical Center of PLA General Hospital Beijing China; ^2^ Hebei North University Zhangjiakou Hebei China; ^3^ Department of Geriatrics The Eighth Medical Center of PLA General Hospital Beijing China; ^4^ Faculty of Medicine and Health Technology Tampere University Tampere Finland

**Keywords:** diabetes mellitus, hyperglycemia, immune response, *Mycobacterium tuberculosis*, tuberculosis

## Abstract

The coexistence of diabetes mellitus (DM) and tuberculosis (TB) presents a significant global burden, with DM being recognized as a major risk factor for TB. This review comprehensively analyzes the immunological aspects of DM‐TB comorbidity, shedding light on the impact of DM on TB pathogenesis and immune responses. It reveals that high blood glucose levels in TB patients contribute to reduced innate immune cell count, compromised phagocytic function, and delayed antigen presentation. These factors ultimately impair the clearance of *Mycobacterium tuberculosis* (MTB) and delay adaptive immune responses. With the interaction between TB and DM, there is an increase in inflammation and elevated secretion of pro‐inflammatory cytokines by immune cells. This exacerbates the inflammatory response and contributes to poor treatment outcomes in TB. Moreover, the review explores the effects of DM on TB prevention, diagnosis, and treatment. It highlights how poor glycemic control, insulin resistance (IR), DM complications, and genetic factors increase the risk of MTB infection in individuals with DM. Additionally, DM‐related immune suppression adversely affects the sensitivity of traditional diagnostic tests for TB, potentially resulting in underdiagnosis and delayed intervention. To mitigate the burden of TB in DM patients, the review emphasizes the need for further research on the mechanisms underlying DM reactivation in latent TB infection (LTBI). It shows how important it is to find and treat LTBI in DM patients as soon as possible and suggests looking into biomarkers that are specific to DM to make diagnosis more accurate.

## Graphical abstract

The coexistence of diabetes mellitus (DM) and tuberculosis (TB) presents a significant global burden, with DM being recognized as a major risk factor for TB. This review comprehensively analyzes the immunological aspects of DM‐TB comorbidity, shedding light on the impact of DM on TB pathogenesis and immune responses. Moreover, the review explores the effects of DM on TB prevention, diagnosis, and treatment. It highlights how poor glycemic control, insulin resistance, DM complications, and genetic factors increase the risk of MTB infection in individuals with DM. Additionally, DM‐related immune suppression adversely affects the sensitivity of traditional diagnostic tests for TB, potentially resulting in underdiagnosis and delayed intervention. To mitigate the burden of TB in DM patients, the review emphasizes the need for further research on the mechanisms underlying DM reactivation in latent TB infection (LTBI). It underscores the importance of early screening and interventions for LTBI in DM patients and suggests the exploration of DM‐LTBI‐specific biomarkers to improve diagnostic accuracy.

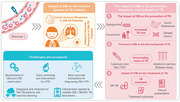

## 1 INTRODUCTION

Diabetes mellitus (DM) is a metabolic disorder characterized by chronic hyperglycemia resulting from defects in insulin secretion and action. DM can cause dysregulation of energy metabolism (carbohydrate, fat, and protein), ultimately leading to multisystem damage.^[^ ^1^ ^]^ It is one of the leading causes of death and disability globally and a significant risk factor for the top two global disease burdens (ischemic heart disease and stroke).^[^ ^2^ ^]^ According to reports, the number of adult DM patients worldwide reached 537 million in 2021, accounting for 10.5% of the global adult population, an increase of 74 million cases compared to 2019.^[^ ^3^ ^]^ This means that one out of every 10 adults is a DM patient. China, India, and Pakistan have the highest number of DM patients among countries.^[^ ^3^ ^]^ Concerningly, the International Diabetes Federation (IDF) projects that the global number of DM patients will reach 643 million by 2030 (11.3% of the global adult population) and 783 million by 2045 (12.2% of the global adult population).^[^ ^3^ ^]^

Previous studies have shown a close correlation between DM and tuberculosis (TB),^[^ ^4^ ^]^ and the increase in the number of DM may make the burden of TB even more severe. TB is a chronic infectious disease caused by *Mycobacterium tuberculosis* (MTB), which infects about a quarter of the world's population and is the leading cause of death from a single infectious agent.^[^ ^5^ ^]^ The World Health Organization's (WHO) latest global TB report shows that in 2022, there were 7.5 million new cases of TB diagnosed in the world, the highest number since the WHO has been counting TB.^[^ ^6^ ^]^ Meanwhile, the number of deaths in that year was 1.3 million, and although the number of global TB deaths decreased by 19% between 2015 and 2022, this figure is well below the 75% reduction by 2025 set as a target by the End TB Strategy.^[^ ^6^ ^]^ It is worth noting that, similar to diabetes, tuberculosis occurs primarily in low‐ and middle‐income countries, with China ranking third globally among countries with a high burden of tuberculosis, accounting for 7.1% of total cases, after India and Indonesia.^[^ ^6^ ^]^

It has been reported that DM is a major risk factor for TB.^[^ ^7^ ^]^ The probability of TB in DM patients is more than three times higher compared to the general population.^[^ ^8^, ^9^ ^]^ DM‐TB patients also exhibit more severe clinical symptoms and more pronounced adverse drug reactions.^[^ ^10^, ^11^ ^]^ In addition, DM increases the risk of TB recurrence and mortality.^[^ ^12^ ^]^ Moreover, studies have found that patients with both TB and DM have a twofold increase in the risk of death and a fourfold increase in the recurrence rate during TB treatment.^[^ ^13^, ^14^, ^15^ ^]^ The increasing number of DM‐TB comorbidities not only poses a serious threat to human life and health but also places a heavy burden on the global healthcare system.^[^ ^6^ ^]^

Although the exact mechanisms by which DM influences susceptibility to TB are not fully understood, recent research has shown that long‐term hyperglycemia in DM patients leads to decreased immune cell numbers and function, thereby increasing the incidence of TB.^[^ ^16^ ^]^ On the one hand, chronic hyperglycemia severely impairs the function of innate immune cells. For example, it may hinder monocyte differentiation into macrophages and dendritic cells, reduce the recruitment, recognition, phagocytosis, and antigen presentation functions of macrophages, and decrease the frequency of dendritic cells and natural killer cells. It also increases the inflammatory response of neutrophils, leading to an exacerbation of bacterial load. To varying degrees, these factors reduce innate immune cells' ability to clear MTB.^[^ ^17^, ^18^ ^]^ On the other hand, chronic hyperglycemia may also delay the activation of adaptive immune cells such as CD4^+^ T cells and CD8^+^ T cells. CD4^+^ T cells play a critical role in anti‐tuberculosis immunity, and their secretion of interferon‐gamma (IFN‐γ) promotes the proliferation of T lymphocytes and activation of macrophages.^[^ ^19^, ^20^, ^21^, ^22^ ^]^ However, under the influence of high blood glucose, the activation of CD4^+^ T cells may be delayed, resulting in a reduced secretion of IFN‐γ.^[^ ^23^, ^24^ ^]^

Therefore, this study aims to explore the impact of DM on the immune systems of TB patients further by consolidating relevant research on DM and TB. Additionally, this study investigates the reasons for the increased susceptibility of DM patients to TB, including poor blood sugar control, insulin resistance (IR), DM complications, and genetic factors. Furthermore, it discusses the effect of diabetes on the sensitivity of immunological tests for TB, such as the tuberculin skin test (TST) and IFN‐γ release assay (IGRA), as well as the association between DM and drug‐resistant TB (DR‐TB) and poor treatment outcomes. Finally, to reduce the risk of TB infection in DM patients, this study proposes the need for further research on the mechanisms underlying the reactivation of latent tuberculosis infection (LTBI) in DM patients and recommends early LTBI screening and intervention for DM patients. Using the ideas of personalized medicine and evidence‐based medicine, future research should look for key biomarkers that can detect DM‐LTBI early on. It should also combine artificial intelligence with diagnostic and therapeutic models and come up with new ways to diagnose and treat DM‐TB.

## 2 IMMUNE CHARACTERISTICS OF DM AND TB PATIENTS

### 2.1 Immune characteristics of DM patients

DM can be classified into different types based on their pathogenic mechanisms, including type 1 diabetes mellitus (T1DM), type 2 diabetes mellitus (T2DM), and other types of DM.^[^ ^25^ ^]^ Among them, T2DM is the most common type, accounting for 90% of all DM cases and showing the strongest association with TB.^[^ ^26^ ^]^ The occurrence and development of both T1DM and T2DM are closely related to alterations in the body's immune system.

#### 2.1.1 Immune characteristics of T1DM

**FIGURE 1:**
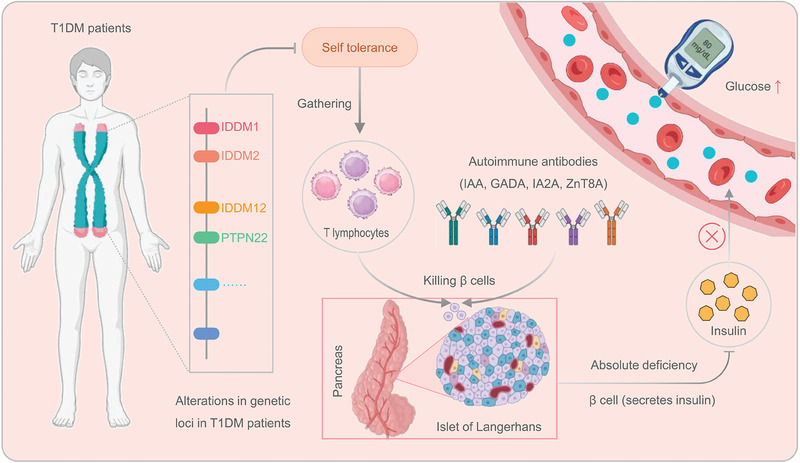
The immune mechanisms of T1DM. In individuals with T1DM, the immune tolerance is disrupted due to genetic alterations in specific loci, such as IDDM1, IDDM2, IDDM12, and PTPN22, among others. The defective immune tolerance leads to the proliferation of T lymphocytes and the targeting of pancreatic islet beta cells by immune cells and autoantibodies (such as IAA, GADA, IA2A, and ZnT8A). This attack destroys pancreatic islet beta cells, leading to a deficiency of insulin and subsequent elevation of blood glucose levels. GADA, glutamic acid decarboxylase; Glu, glutamate; IA2A, insulinoma‐associated antigen; IAA, insulin autoantibodies; T1DM, type 1 diabetes mellitus; ZnT8A, zinc transporter 8.

Research indicates that T1DM is a self‐immune disease closely associated with genetic and environmental factors.^[^ ^27^ ^]^ In genetic studies, it has been found that T1DM is linked to genetic variants in multiple chromosomal regions,^[^ ^28^ ^]^ including the human leukocyte antigen (HLA) region on chromosome 6p21 (IDDM1), the insulin gene region on chromosome 11p15 (IDDM2), the CTLA4 locus on chromosome 2q33 (IDDM12), and the PTPN22 locus on chromosome 1p13.^[^ ^29^ ^]^ Among them, the HLA region on chromosome 6, especially the HLA class II genes, is most strongly associated with T1DM, accounting for approximately 50% of the genetic risk.^[^ ^30^ ^]^ These genetic variations further lead to the breakdown of immune tolerance in the body.^[^ ^31^ ^]^ In addition, previous studies have found the presence of antibodies against antigens such as insulin in individuals, even in the absence of T1DM. However, under normal circumstances, the autoreactive T cells in the peripheral system remain inactive, thereby preventing the onset of the disease.^[^ ^32^ ^]^ However, when immune tolerance in the body is compromised, the immune system fails to respond appropriately to these antibodies, resulting in the expansion of T lymphocytes and their collaboration with autoantibodies targeting insulin (IAA), glutamic acid decarboxylase (GADA), insulinoma‐associated antigen 2 (IA2A), and zinc transporter 8 (ZnT8A).^[^ ^30^ ^]^ This collaboration destroys pancreatic beta cells,^[^ ^32^ ^]^ inadequate insulin secretion, and ultimately, elevated blood glucose levels(Figure 1).^[^ ^33^ ^]^

#### 2.1.2 Immunological characteristics of T2DM

**FIGURE 2:**
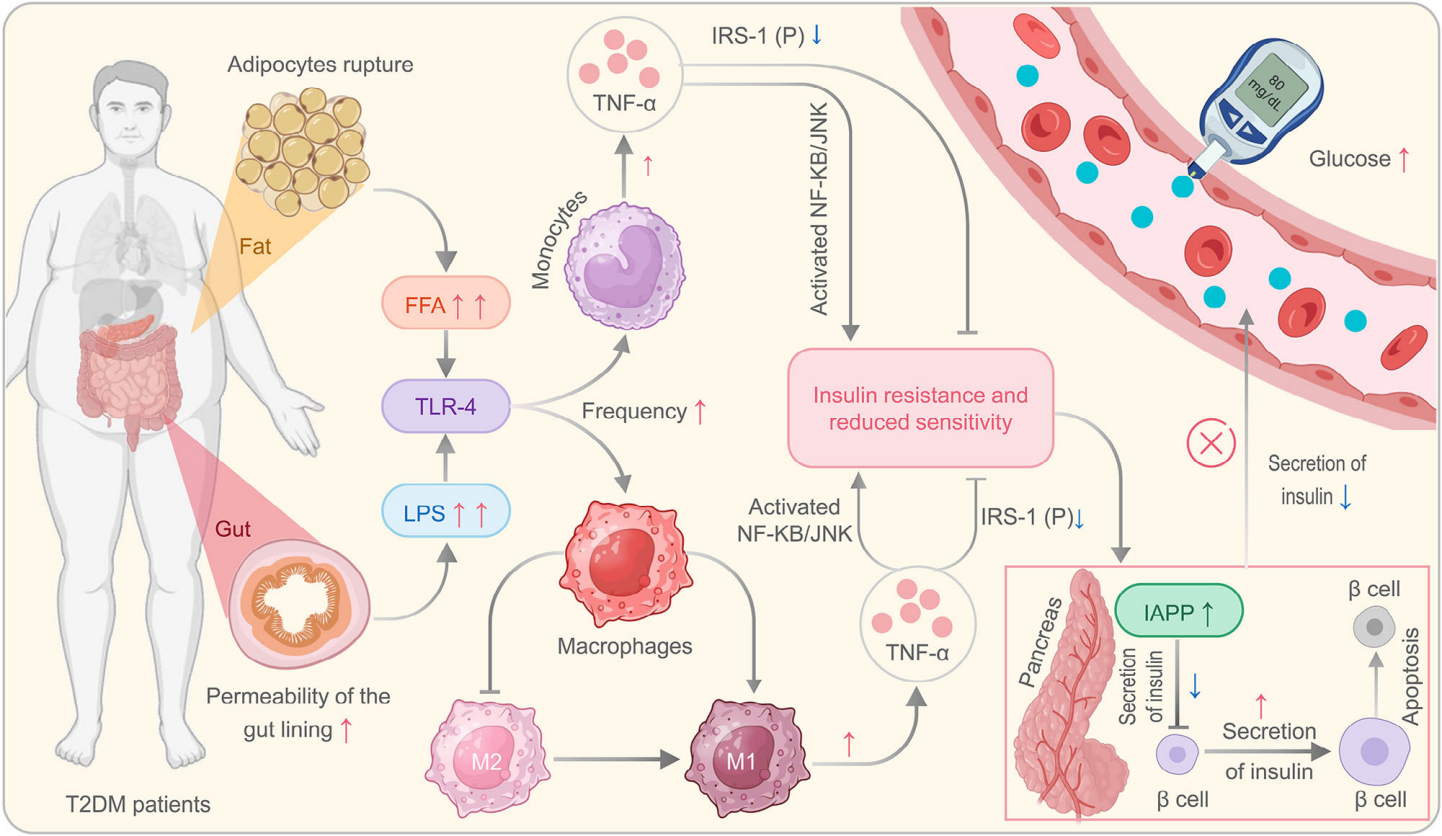
The immune mechanisms of T2DM. In obese individuals with T2DM, enlarged adipocytes can rupture, releasing FFAs into the blood, while a compromised intestinal barrier allows the release of LPS. FFAs and LPS activate macrophages and monocytes through TLR‐4 signaling, converting M2 to M1 macrophages. TNF‐α secretion by M1 cells inhibits insulin signaling by impairing IRS‐1 phosphorylation, leading to insulin resistance. Inflammatory pathways like nuclear factor kappa B (NF‐KB) and c‐Jun N‐terminal kinase (JNK) are activated, reducing tissue sensitivity to insulin. Insulin resistance and reduced sensitivity impede glucose utilization by cells, triggering compensatory secretion of more insulin by pancreatic beta cells. Additionally, IAPP, which inhibits insulin secretion, is also produced. Over time, excessive workload causes beta cell apoptosis, resulting in inadequate insulin secretion and elevated blood glucose levels. FFAs, free fatty acids; Glu, glutamate; IAPP, islet amyloid polypeptide; IRS‐1, insulin receptor substrate 1; LPS, lipopolysaccharide; T2DM, type 2 diabetes mellitus; TLR, toll‐like receptor; TNF‐α, tumor necrosis factor alpha.

T2DM is a chronic metabolic disorder characterized by impaired insulin secretion and IR.^[^ ^34^ ^]^ Previous studies have identified obesity as the primary cause of IR.^[^ ^35^ ^]^ In obese individuals, there is an increase in the number and size of adipocytes, leading to the release of large amounts of free fatty acids (FFAs) into the bloodstream under stress.^[^ ^36^ ^]^ Additionally, obesity is closely associated with impaired intestinal epithelial barrier integrity and increased levels of lipopolysaccharides (LPS), a type of endotoxin found in the gut.^[^ ^37^, ^38^ ^]^ When FFAs and LPS get into the bloodstream, they can set off inflammatory signaling pathways in immune cells, mainly macrophages and monocytes, through toll‐like receptor 4 (TLR4) signaling. This can lead to long‐lasting low‐grade inflammation.^[^ ^37^ ^]^ Surprisingly, in obese populations (e.g., diet‐induced obesity), anti‐inflammatory M2 macrophages can be converted into pro‐inflammatory M1 macrophages.^[^ ^39^, ^40^, ^41^ ^]^ M1 macrophages and monocytes secrete cytokines such as tumor necrosis factor‐alpha (TNF‐α), interleukin‐1 beta (IL‐1β), and IL‐6.^[^ ^42^ ^]^ Among these, TNF‐α plays a crucial regulatory role in insulin signaling and inflammation. In adipocytes, it messes up the phosphorylation of insulin receptor substrate‐1 (IRS‐1). This stops insulin signals from working normally, which leads to IR.^[^ ^43^, ^44^ ^]^ Moreover, TNF‐α can activate inflammatory‐related signaling pathways such as NF‐κB and JNK in adipocytes, promoting the transcription and release of inflammatory factors, further reducing tissue cell sensitivity to insulin.^[^ ^43^, ^44^ ^]^ Due to IR and decreased insulin sensitivity, tissue cells cannot effectively uptake enough glucose for energy production. In response to this metabolic defect, pancreatic beta cells release more insulin into the bloodstream and secrete more islet amyloid polypeptide (IAPP), inhibiting insulin secretion.^[^ ^45^ ^]^ Consequently, the excessive workload placed on pancreatic beta cells leads to significant apoptosis, resulting in insufficient insulin secretion and elevated blood glucose levels (Figure 2).^[^ ^46^ ^]^

### 2.2 Immunological characteristics of TB patients

Although MTB can infect any organ or tissue in the human body except hair and nails, pulmonary tuberculosis (PTB) accounts for nearly 90% of TB cases. MTB mostly gets into the alveoli through the respiratory tract. There, pattern‐recognition receptors on alveolar macrophages help them engulf and destroy MTB, creating phagosomes. With fusion of the phagosome and lysosome, MTB is degraded by acid hydrolases within the phagosome.^[^ ^47^, ^48^ ^]^ Additionally, immature dendritic cells (iDCs), aided by neutrophils, ingest MTB and mature into mature dendritic cells (mDCs) which migrate to lymph nodes.^[^ ^49^ ^]^ DCs break down the antigen and present it along with major histocompatibility complex (MHC) class molecules. This creates antigen complexes that are then shown to T lymphocytes, which activate them.^[^ ^49^ ^]^

T lymphocytes can be further divided into CD4^+^ T lymphocytes and CD8^+^ T lymphocytes based on the surface characteristics of their molecules.^[^ ^50^ ^]^ On the one hand, CD4^+^ T lymphocytes are the primary effector cells of host defense against MTB infection.^[^ ^51^ ^]^ Initially, DC cells take up MTB antigen and present it to initial CD4^+^ T lymphocytes (Th0 cells) via MHC II, and naive CD4^+^ T lymphocytes (Th0 cells) become activated.^[^ ^51^ ^]^ Activated Th0 cells differentiate into various helper T cells, including Th1, Th2, Th17, and regulatory T cells (Treg cells), under the influence of different cytokines.^[^ ^52^ ^]^ (1) Th0 cells differentiate into Th1 cells in the presence of IL‐12 and IFN‐γ. Th1 cells produce multiple cytokines, such as IFN‐γ, IL‐2, and TNF‐α, which activate macrophages and contribute to the clearance of MTB.^[^ ^53^, ^54^ ^]^ (2) Th0 cells can differentiate into Th2 cells in the presence of IL‐2 and IL‐4. Th2 cells activate B lymphocytes and promote their differentiation into plasma cells by secreting cytokines such as IL‐4, IL‐5, and IL‐10, participating in humoral host defense.^[^ ^55^ ^]^ (3) Th0 cells can differentiate into Th17 cells under the influence of cytokines like IL‐6, IL‐21, IL‐23, and TGF‐β. Th17 cells primarily secrete IL‐17, Th17 cells mainly secrete IL‐17, which promotes neutrophil recruitment to the site of infection, and T‐lymphocyte migration to the lung tissue, which plays an important role in the timely fight against MTB infection.^[^ ^56^ ^]^ (4) Th0 cells can also differentiate into Treg cells with immunosuppressive function under the influence of cytokines like IL‐10 and TGF‐β. Treg cells help prevent excessive immune responses and immune‐mediated tissue damage and regulate the balance between Th1, Th2, Th17, and other immune cells to maintain normal immune system function. Therefore, CD4^+^ T lymphocytes differentiate to varying degrees into Th1, Th2, Th17, and Treg cells, playing a crucial role in host defense against MTB infection.

On the other hand, CD8^+^ T lymphocytes are activated into cytotoxic T lymphocytes (CTLs) upon recognition of MTB antigen peptides presented by MHC class I molecules. CTLs secrete perforin and granzymes to kill target cells and release toxic substances, leading to the death of MTB. Additionally, CTLs secrete cytokines such as IFN‐γ and TNF‐α to activate macrophages, enhancing their ability to clear MTB.^[^ ^57^ ^]^

## 3 IMPACT OF DM ON THE IMMUNE SYSTEM OF TB PATIENTS

**FIGURE 3:**
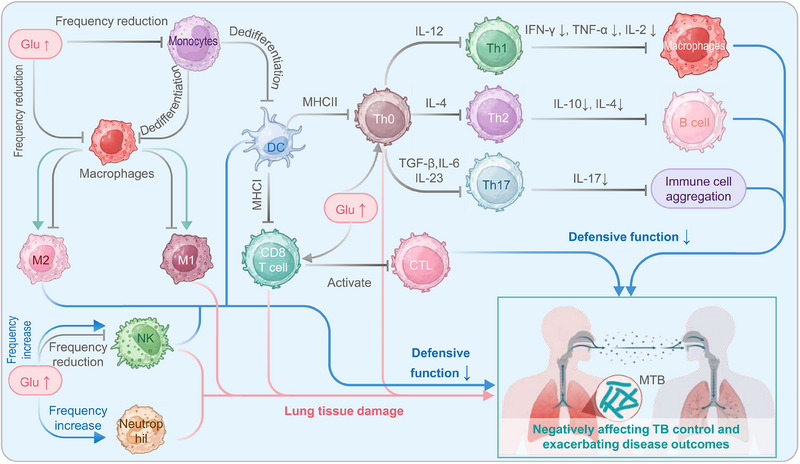
The influence of DM on the immune system of TB patients. On one hand, TB patients' elevated blood glucose levels hinder the differentiation of monocytes into DCs and macrophages. The reduction in DCs leads to a decline in antigen presentation and activation of CD4^+^ T cells (Th0). Consequently, the diminished Th0 differentiation results in decreased secretion of corresponding cytokines, affecting the activation of macrophages and humoral immunity and impairing the recruitment of immune cells. This poses a significant problem for TB patients as they are more susceptible to hyperglycemia. Additionally, high blood glucose levels also impact the polarization of macrophages, enhancing M2 activation and reducing M1 activation and affecting the frequency and cytotoxicity of DCs and natural killer (NK) cells. These effects compromise the ability to clear *Mycobacterium tuberculosis* infection. On the other hand, high blood glucose levels can also lead to increased M1 activation, decreased M2 activation, and increased frequency and activity of NK cells, neutrophils, CD4^+^ T lymphocytes, and CD8^+^ T lymphocytes, contributing to pathological damage in the host. Overall, diabetes disrupts the normal function and frequency of immune cells, negatively impacts TB control, and exacerbates disease outcomes. CTL, cytotoxic T lymphocyte; DC, dendritic cells; DM, diabetes mellitus; Glu, glutamate; IFN, interferon; IL, interleukin; NK cell, natural killer cell; MHC, major histocompatibility complex; TB, tuberculosis; TNF, tumor necrosis factor.

Chronic hyperglycemia can impair the immune system function, leading to delayed activation, diminished recognition and phagocytic function of host immune cells, and reduced secretion of chemokines and cytokines.^[^ ^58^, ^59^ ^]^ This significantly increases the susceptibility of DM patients to TB (Figure 3). Unfortunately, when DM patients are simultaneously infected with MTB, the inflammatory response in the body is also heightened, exacerbating the clinical symptoms and adverse prognosis of the patients.

### 3.1 Innate immune response in DM‐TB patients

Innate immune cells, including monocytes, macrophages, DCs, NK cells, and neutrophils, serve as the first line of defense against pathogen invasion. However, DM commonly affects the number and defensive function of innate immune cells, resulting in increased susceptibility of DM patients to TB and aggravation of TB symptoms in patients (Table 1).^[^ ^17^, ^18^, ^58^, ^60^, ^61^, ^62^, ^63^, ^64^, ^65^, ^66^, ^67^, ^68^, ^69^, ^70^, ^71^, ^72^, ^73^, ^74^, ^75^, ^76^, ^77^, ^78^, ^79^ ^]^

**TABLE 1:** DM affects the innate immune response of TB patients.

Cell type	Effects of DM on immune cells in patients with TB	Ref.
Monocytes	(1) Inhibited differentiation of monocytes into macrophages. (2) Impaired differentiation of monocytes into dendritic cells. (3) Suppressed phagocytic activity of monocytes. (4) Reduced secretion of IL‐1β and IL‐8 due to glibenclamide administration in patients with diabetes mellitus. (5) Altered complement pathway leading to impaired activation of monocyte signaling pathways in patients.	[17, 60, 61, 62, 63, 64, 65]
Macrophages	(1) GPR183 and oxysterol expression decrease, leading to reduced recruitment of macrophages. (2) The recognition function of macrophages toward MTB is impaired. (3) The antigen presentation function of macrophages is decreased. (4) Expression of macrophage receptors and CD14 decreases. (5) Polarization of M2 macrophages increases. (6) Expression levels of HLA‐DR, CD80, and CD86 decrease. (7) M1 pro‐inflammatory response increases. (8) Expression of HIF‐1 regulatory genes is downregulated.	[18, 66, 67, 68, 69, 70, 71, 72, 73, 74]
DC	(1) The frequency of DC is negatively correlated with blood glucose levels. (2) DCs exhibit decreased secretion of IFN‐y. (3) The differentiation and maturation of DCs are hindered.	[62, 75, 76, 77]
Neutrophils	(1) The frequency of neutrophils significantly increases. (2) Neutrophil adhesiveness is impaired. (3) DM can induce NET. (4) Neutrophils secrete decreased levels of IL‐22.	[62, 75, 76, 77]
NK cell	(1) The frequency of NK cells is negatively correlated with fasting blood glucose. (2) The frequency of CD107a in NK cells is decreased. (3) NK cells secrete increased levels of IL‐6. (4) TNF‐a and IL‐17 in NK cells are significantly elevated.	[58, 78, 79]

Abbreviations: DC, dendritic cells; DM, diabetes mellitus; HIF‐1, hypoxia‐inducible factor 1; HLA‐DR, human leukocyte antigen‐DR; IFN, interferon; IL, interleukin; MTB, *Mycobacterium tuberculosis*; NET, neutrophil extracellular traps; NK cell, natural killer cell; TB, tuberculosis.

#### 3.1.1 Monocytes

Monocytes play a crucial role in the innate immune response of the body. When the body is infected with MTB, monocytes can quickly migrate to the lungs and differentiate into macrophages and DCs. They phagocytose, kill MTB, and present relevant antigens to activate other immune cells.^[^ ^80^ ^]^ Additionally, monocytes can secrete various inflammatory regulatory factors, such as TNF‐α, IFN‐γ, and IL‐12. These factors can promote the activation of immune cells and enhance the immune response against TB. Moreover, monocytes are also involved in regulating the immune response of T cells and balancing the Th1/Th2 cell immunity to maintain normal immune function.

However, monocyte immune function is affected to some extent by high blood glucose levels. First, the differentiation of monocytes into macrophages is hindered under high blood glucose conditions.^[^ ^61^ ^]^ The differentiation of monocytes into macrophages requires binding with 1,25‐dihydroxy vitamin D3 (1,25(OH)_2_D_3_) and vitamin D receptor. However, due to IR and increased chemokine receptor CCR2 on monocyte surfaces, DM patients have reduced vitamin D levels, which hinders the differentiation of macrophages.^[^ ^60^, ^61^ ^]^ Studies have also found that high blood glucose conditions hinder the differentiation of monocytes into DCs. The decreased differentiation of DCs can impact their critical role in antigen presentation and phagocytosis, thereby increasing the challenge of resisting MTB infection.^[^ ^17^ ^]^ Second, García et al. demonstrated using fluorescent *Mycobacterium bovis* Bacillus Calmette–Guérin (BCG) that the phagocytic function of monocytes in DM patients is inhibited.^[^ ^62^, ^63^ ^]^ Researchers have also found that in some DM patients, monocytes secrete lower levels of cytokines such as IL‐1β and IL‐8 after administering glimepiride, thereby reducing the phagocytic function of monocytes.^[^ ^64^ ^]^ IL‐1β is crucial for the body's defense against MTB, as it can restrict the excessive production of type I IFN‐γ by increasing the production of cyclooxygenase and helping control MTB.^[^ ^64^ ^]^ Last, studies have found that the interaction between MTB and monocytes is significantly reduced in DM‐TB patients. This may be due to changes in the complement pathway caused by high blood glucose, which affects the downstream activation of monocyte signaling pathways and diminishes the functions of monocytes.^[^ ^65^ ^]^ In summary, under conditions of chronic hyperglycemia, the differentiation frequency and antimicrobial function of monocytes are reduced.

#### 3.1.2 Macrophages

Macrophages are the first line of defense against MTB when it invades the human body.^[^ ^81^ ^]^ On the one hand, macrophages eliminate MTB by recognizing and engulfing the bacteria to form MTB phagosomes, clearing MTB through the acidic environment of lysosomes and releasing antimicrobial substances such as reactive oxygen species (ROS) and nitric oxide.^[^ ^82^ ^]^ On the other hand, macrophages can secrete various cytokines such as TNF, IFN‐γ, and IL‐12.^[^ ^83^ ^]^ These cytokines not only stimulate and regulate the immune response but also activate other immune cells, such as T cells and NK cells, to enhance the antimicrobial activity against MTB.

However, under chronic hyperglycemia conditions, macrophages' response to pathogens is affected in several ways. Previous studies have found that DM affects macrophages' recruitment, recognition, phagocytosis, and antigen presentation functions.^[^ ^18^, ^67^ ^]^ To begin, it has been discovered that people with DM‐TB have lower levels of GPR183 and oxysterol, which impacts the recruitment of macrophages.^[^ ^18^ ^]^ Meanwhile, other studies have also found that under high blood glucose conditions, the recognition function of alveolar macrophages for MTB is impaired, affecting the initiation of the innate immune response.^[^ ^66^ ^]^ Additionally, studies have shown that DM can reduce the antigen presentation function of macrophages, leading to delayed adaptive immune response.^[^ ^67^ ^]^ Second, Martinez et al. found that in DM‐TB mice, the expression of macrophage receptors such as MARCO and CD14 is downregulated, resulting in impaired phagocytic function of alveolar macrophages.^[^ ^68^ ^]^ Similarly, Verma et al. used animal models to show that when the host's blood glucose level rises, both in TB and LTBI, macrophages are less able to get rid of bacteria.^[^ ^69^ ^]^ Last, studies have shown that in the high‐glucose pulmonary microenvironment, macrophages polarize into M2‐type macrophages and significantly upregulate the expression of MMP9 and CD169, reducing macrophages' phagocytic and antimicrobial capabilities.^[^ ^70^ ^]^ Additionally, Kewcharoenwong et al. found that glimepiride can promote M2 polarization, impairing macrophages' bactericidal ability and increased susceptibility to TB in the host.^[^ ^71^ ^]^

In addition to affecting the function of macrophages, DM also increases their inflammatory response and aggravates tissue damage in the body. Lopez et al. found through a controlled study that the activation status of macrophages changes in TB‐MB patients, with a decrease in the expression of HLA‐DR, CD80, and CD86, and a significant increase in Programmed Death‐Ligand 1 (PD‐L1) expression.^[^ ^72^, ^73^ ^]^ Elevated levels of PD‐L1 are immunosuppressive molecules for T cells, and their increase can inhibit the immune function of T cells.^[^ ^72^, ^73^ ^]^ Furthermore, some studies suggest that DM may lead to polarization of M1 macrophages, possibly due to abnormal accumulation of advanced glycation end products (AGEs) caused by high blood glucose.^[^ ^84^, ^85^, ^86^ ^]^ AGEs can induce M1 polarization of macrophages through hypoxia‐inducible factor‐1a (HIF‐1a), pyruvate dehydrogenase kinase (PDK) signaling pathways, and mitogen‐activated protein kinase (MAPK) signaling pathways.^[^ ^87^, ^88^ ^]^ Worrisomely, in DM patients combined with TB, the pro‐inflammatory response of M1 macrophages may form a vicious cycle, leading to aggravated body inflammatory response and ultimately resulting in lung tissue damage.^[^ ^72^, ^73^ ^]^ Additionally, Zhao et al. found that the expression of HIF‐1‐regulated genes in bone marrow‐derived macrophages (BMMs) increases in TB mice, which helps control the bacterial load.^[^ ^74^ ^]^ However, in DM‐TB mice, high blood glucose hinders the expression of HIF‐1‐regulated genes, impairing the protective immune response and the ability of BMMs to control bacteria.^[^ ^74^ ^]^

In summary, DM affects macrophages' recruitment, recognition, phagocytosis, and antigen presentation functions. Additionally, it increases the inflammatory response of macrophages, further exacerbating tissue damage in the body.

#### 3.1.3 DCs

DCs are important bridging cells between innate and adaptive immunity, and they are known as the most effective antigen‐presenting cells (APCs). Upon MTB infection, iDCs capture a large number of pathogens and migrate to lymph nodes, where they gradually mature and differentiate into mDCs with significantly enhanced antigen presentation capability, ultimately efficiently activating adaptive immune responses.^[^ ^49^, ^89^ ^]^

While DCs play a critical role in activating adaptive immune responses, a high blood glucose state may impair their functions. To explore the impact of high blood glucose on DCs, researchers conducted experiments by comparing subgroups of white blood cells between TB and DM‐TB patients.^[^ ^90^ ^]^ The study results demonstrated a significant decrease in DC frequency in DM‐TB patients, accompanied by delayed activation, migration, and antigen presentation of DCs.^[^ ^90^ ^]^ Moreover, it was found that there is a negative correlation between DC frequency and high blood glucose levels, independent of bacterial load.^[^ ^91^ ^]^ After standardized TB treatment, the DC frequency in DM‐TB patients showed a reversal, further confirming that high blood glucose is a key factor contributing to the decreased frequency of DCs in TB patients.^[^ ^91^ ^]^

Interestingly, multiple studies have found that without affecting the proliferation of CD4^+^ T cells, high blood glucose levels lead to a reduction in IFN‐γ production by CD4^+^ T cells. This may be due to the decreased MHC‐II expression level on APCs (such as macrophages and dendritic cells) caused by high blood glucose concentration, which affects antigen presentation and fails to activate appropriate T cells, resulting in decreased IFN‐γ production and ultimately impairing the immune response against MTB.^[^ ^92^, ^93^, ^94^ ^]^ Additionally, another study found that high blood glucose concentration can reduce the differentiation of monocytes into dendritic cells, increase the production of ROS, and activate the Wnt/B‐catenin and p38 mitogen‐activated protein kinase (MAPK) signaling pathways, which hinder the differentiation and maturation of dendritic cells.^[^ ^17^ ^]^ Furthermore, quercetin can inhibit the effect of the p38 MAPK signaling pathway, thereby counteracting the immunosuppressive effect induced by high blood glucose.^[^ ^17^ ^]^

#### 3.1.4 Neutrophils

Neutrophils, the most abundant white blood cells in the human bloodstream, account for 50% to 70% of the white blood cell count and serve as the first line of defense in the body's innate immune response. Neutrophils can phagocytose and kill MTB through oxidative and non‐oxidative mechanisms. In the oxidative pathway, the release of ROS by neutrophils is the major factor in killing intracellular MTB.^[^ ^95^ ^]^

Neutrophils are closely associated with inflammatory processes in the body. When patients with DM develop TB, neutrophils in the body often exhibit excessive inflammatory responses, exacerbating the overall inflammatory reaction. Multiple studies have indicated that neutrophils are crucial factors in the disease progression in DM‐TB patients, and neutrophil infiltration and the formation of neutrophil extracellular traps (NETs) play central roles in the adverse outcomes of TB and T2DM.^[^ ^96^ ^]^ To understand the key role of neutrophils in DM‐TB patients, Medina et al. conducted a comparative study on 120 Indian subjects, including TB patients, DM patients, DM‐TB patients, and healthy individuals. They found that the absolute neutrophil count was higher in DM‐TB patients compared to other subgroups.^[^ ^75^ ^]^ This suggests that the neutrophil‐associated inflammatory response is elevated in DM‐TB patients, possibly contributing to increased bacterial burden and worsening clinical symptoms. Similarly, Garcia et al. observed an increased absolute neutrophil count in DM‐TB patients, but they also found impaired adhesiveness of neutrophils in DM patients, leading to reduced phagocytic function.^[^ ^62^ ^]^ Furthermore, Wong et al. discovered that high blood glucose levels can induce neutrophils to release NETs, causing hindered tissue healing and exacerbating tissue damage, ultimately leading to chronic inflammation in DM patients.^[^ ^76^ ^]^ This may also contribute to worsened pathological manifestations in DM patients after contracting TB. Additionally, Tripathi et al. found in a mouse model study that mice with both TB and high blood glucose levels exhibited significantly reduced secretion of IL‐22, a cytokine produced by neutrophils, resulting in excessive inflammatory responses and exacerbated tissue damage.^[^ ^77^ ^]^

Therefore, although neutrophils actively participate in inflammatory responses in a high blood glucose state, they also inflict inevitable damage to the body, increasing the susceptibility of DM patients to tuberculosis. Furthermore, the high blood glucose environment disrupts the survival microenvironment of neutrophils, leading to decreased bactericidal function and ultimately weakening the body's defense against MTB.

#### 3.1.5 NK cells

NK cells are a unique subset of lymphocytes.^[^ ^97^ ^]^ They originate from the bone marrow and circulate in the blood, becoming activated through the stimulation of cytokines or target cells. Once activated, NK cells can kill target cells by releasing granules containing enzymes and perforins, and they secrete cytokines such as IFN‐γ, TNF‐α, IL‐17, and IL‐22 to regulate immune responses.^[^ ^98^ ^]^ NK cells serve as sentinels in the immune system's defense against MTB and rapidly participate in immune responses, alerting the host upon invasion by pathogenic bacteria.^[^ ^99^, ^100^ ^]^

To understand the impact of high blood glucose on NK cells, Wei et al. analyzed blood samples from 327 patients, including those with DM‐TB and non‐DM‐TB (NDM‐TB). They found a negative correlation between the number of NK cells in DM‐TB patients and fasting blood glucose (FBG) levels.^[^ ^58^ ^]^ The number of NK cells decreased significantly with increasing FBG levels, suggesting an inhibitory effect of high blood glucose on NK cells.^[^ ^58^ ^]^ Similarly, another study found that the expression frequency of lysosome‐associated membrane protein 1 (CD107a) in NK cells from DM‐TB patients was significantly decreased. CD107a is a marker of NK cell degranulation, and its expression level is closely related to NK cell cytotoxic activity. This suggests that high blood glucose may lead to a decrease in the bactericidal function of NK cells.^[^ ^78^ ^]^

In addition to affecting the frequency and function of NK cells, DM also plays a significant role in inflammation. For example, Cheekatla et al. found in a DM‐TB mouse model study that the interaction between NK cells and CD11c^+^ cells promotes the secretion of IL‐6, leading to an increase in the levels of pro‐inflammatory factors and reduced survival rates in DM‐TB mice, exacerbating tissue damage.^[^ ^79^ ^]^ Moreover, a comparative study by Kumar et al. found that the expression levels of TNF‐α and IL‐17, pro‐inflammatory cytokines secreted by NK cells, were significantly elevated in DM‐TB patients, potentially leading to excessive inflammatory responses and tissue damage.^[^ ^78^ ^]^

In summary, in a state of high blood glucose, the number, function, and activity of NK cells are affected, weakening the body's ability to clear bacteria and potentially leading to increased pro‐inflammatory responses and pathological damage.

### 3.2 Adaptive immune response in TB‐DM patients

The body's immune response against MTB primarily relies on adaptive immunity, initiated by activating innate immune cells for antigen presentation. Only T and B cells can produce particular responses to exogenous antigens, thus initiating acquired immunity. T cells can recognize and accept different antigen peptides presented by APCs through their antigen recognition receptors. CD8^+^ T cells mainly recognize antigen peptides presented by MHC class I molecules, while CD4^+^ T cells mainly recognize antigen peptides presented by MHC class II molecules. While defending against MTB, the functionality of adaptive immune cells can be affected by the high blood glucose state (Table 2).^[^ ^24^, ^78^, ^90^, ^94^, ^101^, ^102^, ^103^, ^104^, ^105^, ^106^, ^107^, ^108^, ^109^, ^110^, ^111^, ^112^, ^113^ ^]^

**TABLE 2:** Effect of DM on adaptive immunity in TB patients.

Cell type	Different perspectives	Effects of DM on immune cells in patients with TB	Ref.
CD4^+^ T cell	Pro‐inflammatory factors ↓ Anti‐inflammatory factors ↑ (Immune response ↓)	(1) IFN‐γ and IL‐12 levels reduced in DM‐TB mice. (2) Production of IFN‐γ cytokine, IL‐10, and IL‐12 cytokine was lower in patients with DM‐TB. (3) IFN‐γ production was reduced at the time points of diagnosis, 30 days’ treatment, and 6 months’ treatment in the DM (p)‐TB group. (4) % of Th2 (CD4^+^IL‐4^+^, CD4^+^IL‐6^+^, and CD4^+^IL‐10^+^) T cells increased in DM‐TB patients. % of CD4^+^IL‐17a T cells increased in DM‐TB patients. (5) IL‐10 production was reduced in patients with DM‐TB compared to NDM‐TB. (6) Th2 (IL‐4, IL‐10) T cells increased in DM‐TB patients.	[24, 101, 102, 103, 105, 106]
	Pro‐inflammatory factors ↑ Anti‐inflammatory factors ↓ (Tissue injury ↑)	(1) Th1 (IFN‐γ, IL‐2, TNF‐α) and Th17 (IL‐17) T cells increased in DM‐TB patients. (2) TNF‐α, granulocyte monocyte–CSF, IFN‐γ, IL‐2, and IL‐1β levels increased in patients with DM‐TB and elevated HbA1c levels. (3) Th1 (TNF‐α, CX3CR1) and Th17 (IL‐17) responses increased in TB with Stable/increased‐HbA1c patients. Th2 (IL‐10) responses decreased in TB with Stable/increased‐HbA1c patients. (4) Th1 (IFN‐γ [PPD and WCL], TNFα [PPD and WCL], and IL‐2 [PPD]) cytokine genomes increased in LTBI‐DM/ LTBI‐PDM. Th17 (IL‐17A, IL‐17F, and IL‐22 [PPD and/or WCL]) cytokine genomes increased in LTBI‐DM/ LTBI‐PDM.	[94, 104, 107, 108, 109]
CD8^+^ T cell		(1) CD8^+^ T cells secrete increased levels of pro‐inflammatory factors such as IFN‐γ, IL‐17, and IL‐2. (2) Expression of cytotoxic markers is significantly reduced. (3) Central memory CD8^+^ T cells are positively correlated with blood glucose levels, while naïve CD8^+^ T cells are negatively correlated. (4) The frequency of CD8^+^ T cells increases.	[78, 110, 111]
B cell		(1) There is an increase in the frequency of memory B cells and a decrease in the frequency of naïve B cells. (2) Elevated blood glucose levels impair humoral immune function. (3) Apoptosis of B cells is suppressed by high blood glucose levels.	[90, 112, 113]

Abbreviations: DM, diabetes mellitus; IFN, interferon; IL, interleukin; TB, tuberculosis.

#### 3.2.1 CD4^+^ T cells

CD4^+^ T cells play a central role in adaptive immunity and can be divided into subsets based on the cytokines they secrete, such as Th1, Th2, Th17, and Treg cells. Th1 and Th17 cells secrete pro‐inflammatory cytokines, while Th2 and Treg cells secrete anti‐inflammatory cytokines.^[^ ^114^ ^]^ First, Th1 cells secrete cytokines such as IFN‐γ, TNF‐α, IL‐2, and IL‐12, which can promote the activation of macrophages and enhance their bactericidal activity.^[^ ^115^ ^]^ Second, Th2 cells secrete cytokines such as IL‐4, IL‐5, IL‐6, and IL‐10, mainly activating the humoral immune system, including B cells, eosinophils, and basophils.^[^ ^116^ ^]^ Last, Th17 cells primarily secrete IL‐17, which can induce neutrophil infiltration and promote the production of IL‐12 and IFN‐γ in dendritic cells, mediating Th1 responses. This indicates the important role of IL‐17 in regulating innate immune responses and the defense and clearance of pathogens until adaptive immune cells are activated and enter the infection site.^[^ ^117^, ^118^, ^119^ ^]^ Additionally, Treg cells secrete anti‐inflammatory cytokines and can regulate the pro‐inflammatory function of Th1 cells, preventing excessive inflammatory responses and protecting against tissue damage.^[^ ^120^, ^121^ ^]^

Early studies yielded different views on the relevance of different subsets of CD4^+^ T cells in the anti‐tuberculosis effect. On the one hand, some research suggests that CD4^+^ T cells from DM‐TB patients exhibit decreased pro‐inflammatory cytokines and increased anti‐inflammatory cytokines, which suppress immune function. For example, it has been found that DM affects the production of Th1 cells and leads to a decrease in immune function. Yamashiro et al. found in a DM mouse model induced by streptozotocin that Th1 cells were significantly suppressed in DM mice, and the synthesis of IL‐12 was also reduced when they were co‐infected with MTB.^[^ ^101^ ^]^ However, Th1 cells primarily produce IFN‐γ, and IL‐12 can promote the production of IFN‐γ by innate immune cells, which is a major effector cytokine for resistance against MTB. Thus, when IFN‐γ secretion is reduced, immune function may be impaired, ultimately increasing the risk of MTB infection.^[^ ^24^, ^101^, ^122^ ^]^ Similarly, in a comparative study by Tsukaguchi et al., it was found that the secretion levels of IFN‐γ in DM‐TB patients were significantly lower than in NDM‐TB patients, and this decrease was more pronounced in poorly controlled DM‐TB patients, indicating a negative correlation between IFN‐γ production and DM control level.^[^ ^24^ ^]^ Furthermore, to further understand the effect of glycemic control on IFN‐γ secretion, Tsukaguchi et al. measured the levels of IFN‐γ during anti‐TB treatment in patients.^[^ ^102^ ^]^ They found that in TB patients with well‐controlled blood glucose who had T2DM, the levels of IFN‐γ secretion returned to levels similar to those of the control group after 6 months of anti‐TB treatment, while the production of IFN‐γ in TB patients with poor glycemic control remained decreased, further confirming the relationship between the inhibition of IFN‐γ secretion and the high blood glucose state.^[^ ^102^ ^]^ Similarly, the study by Stalenhoef et al. suggests that IFN‐γ secretion is reduced in patients with DM‐TB.^[^ ^105^ ^]^ The balance between Th1 and Th2 cells is crucial in the anti‐tuberculosis immune response.^[^ ^123^ ^]^ Wang et al. found that both T2DM and TB patients exhibited an increase in Th2 differentiation, and Th2 cells secreted anti‐inflammatory cytokines, which may lead to the suppression of anti‐TB immunity.^[^ ^103^ ^]^ Not coincidentally, the findings of Attiyah et al. suggest an increase in cytokine secretion by Th2 cells.^[^ ^106^ ^]^ Therefore, the high blood glucose state may decrease IFN‐γ secretion by Th1 cells, an increase in Th2 differentiation, and a subsequent reduction in immune capacity, ultimately affecting the ability to clear bacteria.

On the other hand, some studies have observed an increase in pro‐inflammatory cytokines and a decrease in anti‐inflammatory cytokines in DM‐TB patients, which may lead to increased MTB loads, severe pathological tissue damage, and an inability to mount effective immune responses. First, to determine the role of Th1 and Th17 cells in DM‐TB patients, Kumar et al. found in their study that Th1 and Th17 cell frequencies were elevated in DM‐TB patients compared to NDM‐TB patients, suggesting that this may lead to increased immunopathology in DM‐TB patients.^[^ ^94^ ^]^ They then took the next step of validation to further understand the impact of cytokine levels in DM‐TB patients.^[^ ^107^ ^]^ They found that Th1 (IFN‐γ, TNF‐α, and IL‐2) and Th17 (IL‐17A) cytokine levels were significantly elevated in patients with DM‐TB.^[^ ^107^ ^]^ Thus their data suggest that T2DM underlying chronic inflammation may contribute to increased pathology and poor control of TB infections.^[^ ^107^ ^]^ Similarly, Restrepo et al. conducted immune stimulatory tests on whole blood samples from DM‐TB patients and TB patients and found that Th1 cells in DM‐TB patients exhibited stronger cytokine responses than NDM‐TB patients.^[^ ^104^ ^]^ Restrepo et al. also mentioned that mice with DM alone lagged in IFN‐γ production but exhibited enhanced Th1 cell activity in combination with TB, which may be related to the increase in late‐stage glycosylation end‐products in patients with DM as well as the increased antigenic stimulation following MTB infection.^[^ ^104^ ^]^ In addition, unlike the previous studies, Krause et al. looked at the relationship between hemoglobin A1c (HbA1c) and immune cell changes before and after TB treatment.^[^ ^108^ ^]^ They found that compared to TB patients with decreased HbA1c, patients with stable/elevated HbA1c had elevated expression of TNF‐α, CX3CR1, and IL‐17 and decreased expression of IL‐10.^[^ ^108^ ^]^ The corresponding Th1 and Th17 of these cytokines were then upregulated, as well as Th2 downregulated.^[^ ^108^ ^]^ Studies have shown that glycemic control is associated with an increased pro‐inflammatory state in TB patients.^[^ ^108^ ^]^ Moreover, most have found an effect of DM on the immune response to TB, and less attention has been paid to the effect of DM on LTBI.^[^ ^109^ ^]^ A study by Kathamuthu et al. found that in MTB stimulation, compared to the LTBI‐NDM group, the levels of cytokines secreted by Th1 (IFN‐γ, TNF‐α, and IL‐2) and Th17 (IL‐17A, IL‐17F, and IL‐22) cells were significantly higher in both the LTBI‐DM and LTBI‐PDM groups.^[^ ^109^ ^]^ The study demonstrated that DM would also lead to an increased inflammatory response in LTBI.^[^ ^109^ ^]^

We reviewed and analyzed the studies supporting the two different views, including subjects, sample size, tissues, stimuli, and experimental methods (Table 3).^[^ ^24^, ^94^, ^101^, ^102^, ^103^, ^104^, ^105^, ^106^, ^107^, ^108^, ^109^ ^]^ We found that most of the studies supporting the view that “CD4^+^ T cells in DM‐TB patients exhibit reduced pro‐inflammatory cytokines and increased anti‐inflammatory cytokines, thereby suppressing immune function” were from Asian regions, such as Japan, China, and Indonesia, and the stimuli used were mainly purified protein derivatives (PPD) and BCG. In contrast, studies supporting the idea that “DM‐TB patients have increased pro‐inflammatory cytokines and decreased anti‐inflammatory cytokines, which may lead to an increased MTB load, severe pathological tissue damage, and inability to generate an effective immune response” have been conducted in Europe, America, Southeast Asia, and South Africa, with stimulants mainly based on CFP‐10 and ESAT‐10. The stimulants used were mainly CFP‐10 and ESAT‐6. Despite the existence of these differences, we should be cautious in analyzing the above data because the different experimental results may also come from other unknown confounding factors.

**TABLE 3:** The effect of high blood glucose levels on pro‐inflammatory and anti‐inflammatory cytokines in CD4^+^ T cell subgroups in DM‐TB patients.

Perspectives	Authors, country, year	Study Objects	Groups and simple size	Tissue	Stimulant	Experimental methods	Results^a^	Ref.
Pro‐inflammatory factors↓ Anti‐inflammatory factors↑	Yamashiro et al, Japan, 2005	Mice	DM‐TB, *n *= NA NDM‐TB, *n *= NA	Lung, liver, spleen	PPD	ELISA	(1) IFN‐γ level reduced in the liver and spleen of DM‐TB mice. (2) IL‐12 level reduced in DM‐TB mice's lungs, spleen, and liver. (3) IL‐4 level increased in the lungs and livers of DM‐TB mice.	[101]
	Tsukaguchi et al, Japan, 1997	Human	DM‐TB, *n *= 10 NDM‐TB, *n *= 10 HC:10	PBMC	BCG	ELISA	Production of IFN‐γ cytokine, IL‐10 cytokine, and IL‐12 cytokine was lower in patients with DM‐TB.	[24]
	Tsukaguchi et al, Japan, 2002	Human	DM (p)‐TB and DM (g)‐TB, *n *= 20 NDM‐TB, *n *= 14 HC: *n *= 10	PBMC	BCG	ELISA	(1) IFN‐γ production was reduced at the time points of diagnosis, 30 days’ treatment, and 6 months’ treatment in the DM (p)‐TB group. (2) IFN‐γ production was not significantly different between the DM (g)‐TB and the NDM‐TB groups.	[102]
	Wang et al, China, 2018	Human	DM‐TB, *n *= 23 NDM‐TB, *n *= 23	Whole blood	ESAT‐6, CFP‐10	Flow cytometry	(1) % of Th1 (CD4^+^IFN‐γ^+^, CD4^+^IL‐2^+^, and CD4^+^IFN‐α^+^) T cells showed no significance between two groups. (2) % of Th2 (CD4^+^IL‐4^+^, CD4^+^IL‐6^+^, and CD4^+^IL‐10^+^) T cells increased in DM‐TB patients. (3) % of CD4^+^IL‐17a T cells increased in DM‐TB patients.	[103]
	Stalenhoef et al, Indonesia, 2008	Human	DM‐TB, *n *= 23 DM, *n *= 32 NDM‐TB, *n *= 34 HC, *n *= 36	Whole blood	MTB, LPS, PHA,	ELISA	(1) IL‐10 production is reduced in patients with DM‐TB compared to NDM‐TB. (2) IFN‐γ production was not significantly different between the DM‐TB and NDM‐TB groups, but was significantly lower than that in the DM and HC groups.	[105]
	Attiyah, Kuwait, 2009	Human	DM‐TB, *n *= 11 NDM‐TB, *n *= 18 HC, *n *= 20	PBMC	MTB, BCG, peptide pools of RD1, RD4, RD6, and RD10	Flow cytometry	(1) Th2 (IL‐4, IL‐10) T cells increased in DM‐TB patients. (2) PBMC stimulated by BDG, MTB, Th1 (IFN‐γ) T cells showed no significant difference between DM‐TB and NDM‐TB groups, but when stimulated by RDs, IFN‐γ levels were higher in the DM‐TB group than in NDM‐TB and HC groups. (3) Th1:Th2 cytokine ratios (IFN‐γ:IL‐4, IFN‐γ:IL‐5, IFN‐γ:IL‐10, TNF‐β:IL‐4, TNF‐β:IL‐5, and TNF‐β:IL‐10) were lower in DM‐TB patients than NDM‐TB patients and HC subjects.	[106]
Pro‐inflammatory factors ↑ Anti‐inflammatory factors ↓	Kumar et al, India, 2013	Human	DM‐TB, *n *= 22 NDM‐TB, *n *= 22	Whole blood	PPD, ESAT‐6, CFP‐10	ELISA	(1) Frequencies of Th1 (IFN‐γ, IL‐2, TNF‐α) T cells increased in DM‐TB patients. (2) Th17 (IL‐17) T cell frequency increased in DM‐TB patients.	[94]
	Kumar et al, India, 2013	Human	DM‐TB, *n *= 44 NDM‐TB, *n *= 44	Whole blood	ESAT‐6, CFP‐10, TB 7.7	ELISA	(1) Th1 (IFN‐γ, TNF‐α) T cell levels increased in DM‐TB patients. (2) Th17 (IL‐17) T cell levels increased in DM‐TB patients. (3) Th2 (IL‐10) T cell levels increased in DM‐TB patients.	[107]
	Restrepo et al, United States, Mexico, 2008	Human	DM‐TB, *n *= 29 NDM‐TB, *n *= 37	Whole blood	PPD, SEB	ELISA	TNF‐α, granulocyte monocyte–CSF, IFN‐γ, IL‐2, and IL‐1β levels increased in patients with DM‐TB and elevated HbA1c levels.	[104]
	Krause, South Africa, 2023	Human	TB with Decreased‐HbA1c, *n *= 46 TB with Stable/increased‐HbA1c, *n *= 16	PBMC	Mitogens PMA, ionomycin, MTB peptides (MTB300)	ELISA	(1) Th1 (TNF‐α, CX3CR1) responses increased in TB with stable/increased‐HbA1c patients. (2) Th17 (IL‐17) responses increased in TB with stable/increased‐HbA1c patients. (3) Th2 (IL‐10) responses decreased in TB with stable/increased‐HbA1c patients.	[108]
	Kathamuthu, United States, 2022	Human	LTBI‐DM, *n *= 20 LTBI‐PDM, *n *= 20 LTBI‐NDM, *n *= 20	PBMC	PPD, WCL, phorbol 12‐myristate 13‐acetate, ionomycin	Flow cytometry	(1) Th1 (IFN‐γ [PPD and WCL], TNFα [PPD and WCL], and IL‐2 [PPD]) cytokine genomes increased in LTBI‐DM/LTBI‐PDM. (2) Th17 (IL‐17A, IL‐17F, and IL‐22 [PPD and/or WCL]) cytokine genomes increased in LTBI‐DM/LTBI‐PDM. (3) Cytokine toxicity markers (PFN, GZE B, and GNLSN [PPD and WCL]) decreased in LTB‐DM/LTB‐PDM.	[109]

^a^ The results are derived from the measurement of immune cells and cytokines after stimulation with the stimulants.

Abbreviations: BCG, Bacillus Calmette–Guerin; CFP‐10, culture filtrate protein‐10; DM, diabetes mellitus; ESAT‐6, early secreted antigen‐6; HbA1c, glycated hemoglobin A1c; IFN, interferon; IL, interleukin; LPS, lipopolysaccharide; LTB, latent TB; MTB, *Mycobacterium tuberculosis*; NDM, non‐DM; PBMC, peripheral blood mononuclear cells; PDM, pre‐DM; PPD, purified protein derivative; RD, region deleted; SEB, staphylococcal enterotoxin B; TB, tuberculosis; WCL, whole‐cell lysate.

#### 3.2.2 CD8^+^ T cells

CD8^+^ T cells, also known as CTLs, play a crucial role in the host defense against MTB infection.^[^ ^124^ ^]^ CTLs are capable of releasing perforin, granzymes, and cytokines to counteract pathogens. First, perforin can destroy MTB‐infected macrophages, while granzyme B can enter these damaged cells and kill MTB.^[^ ^125^, ^126^ ^]^ Second, CD8^+^ T cells can secrete cytokines such as IFN‐γ, IL‐17, TNF, IL‐10, IL‐2, and TGF‐β, which promote inflammatory responses to combat MTB infection.^[^ ^127^, ^128^, ^129^, ^130^ ^]^

Unfortunately, DM often affects the normal immune function of CD8^+^ T cells. Kumar et al. found that in DM‐TB patients, the levels of pro‐inflammatory cytokines secreted by CD8^+^ T cells, including IFN‐γ, IL‐17, and IL‐2, were elevated, while the expression of cytotoxic markers perforin, granzyme B, and CD107a on CD8^+^ T cells was significantly reduced.^[^ ^78^ ^]^ With the increase in the secretion of pro‐inflammatory cytokines and the decrease in the ability to clear bacteria by CD8^+^ T cells, the body's resistance is lowered, leading to exacerbation of tissue pathological damage. Additionally, to determine the impact of hyperglycemia on the distribution of CD4^+^ and CD8^+^ T cell subsets in PTB patients, Kumar et al. observed the relationship between CD4^+^ and CD8^+^ T cell subsets and FBG or glycated HbA1c levels in DM‐TB patients.^[^ ^110^ ^]^ They found that the frequency of central memory CD8^+^ T cells was significantly positively correlated with FBG and HbA1c levels, while the frequency of naïve CD8^+^ T cells showed a significant negative correlation with FBG and HbA1c levels.^[^ ^110^ ^]^ This indicates that DM has a certain influence on the frequency of CD8^+^ T cells. Similarly, to investigate the susceptibility of T2DM patients to tuberculosis pneumonia and non‐tuberculosis pneumonia, researchers measured the counts of CD4^+^ and CD8^+^ cells in whole blood, and the results showed that in patients with tuberculosis pneumonia complicated by DM, the counts of CD4^+^ and CD8^+^ cells were significantly increased, while in patients with non‐tuberculosis pneumonia complicated by DM, the counts of CD4^+^ and CD8^+^ cells were significantly decreased.^[^ ^111^ ^]^ This suggests that the interaction between TB and DM leads to an increase in the frequency of CD8^+^ T cells, which may further induce an increase in inflammatory reactions, resulting in worsening of tissue damage.

In summary, DM may decrease the expression of cytotoxic markers in CD8^+^ T cells, thereby weakening the body's ability to clear MTB. Additionally, the increase in the frequency of CD8^+^ T cells and the increase in the secretion of pro‐inflammatory cytokines may also contribute to pathological damage in the body.

#### 3.2.3 B cells

B cells primarily participate in the body's specific humoral immune response. They can exert anti‐tuberculosis immune effects through various mechanisms, including producing antibodies against pathogens, antigen presentation, secretion of cytokines, and influencing leukocyte cytotoxic mechanisms.^[^ ^131^, ^132^ ^]^ Additionally, B cells are essential components of tuberculosis granulomas.^[^ ^133^ ^]^ Currently, there is limited research on the impact of DM on B cell‐mediated anti‐TB immunity. However, the significant role of B cells should not be neglected. Kumar et al. investigated the impact of DM‐TB on leukocyte subset frequencies and identified the influence of DM‐TB on leukocytes.^[^ ^90^ ^]^ The study results showed that DM did not affect the memory B cell subset, but in early‐stage DM combined with TB, there was an increased frequency of classical memory B cells, and in active or latent TB combined with DM, there was an increased frequency of activated memory B cells and atypical memory B cells, while the frequency of naive B cells decreased.^[^ ^90^ ^]^ These results indicate that in DM‐TB patients, the immune response of B cells is to some extent affected, but the specific impact on overall immune function remains unclear. Furthermore, Gholamreza et al. found that hyperglycemia, through non‐enzymatic glycation and covalent glycation of proteins, may affect the biological function of immunoglobulins and impair humoral immunity.^[^ ^112^ ^]^ Additionally, studies have suggested that hyperglycemia might inhibit apoptosis of B cells.^[^ ^113^ ^]^

In summary, DM has a certain impact on TB patients' B‐cell immune response, but the specific mechanisms are yet to be determined, and further research is needed.

## 4 THE IMPACT OF DM ON THE PREVENTION, DIAGNOSIS, AND TREATMENT OF TB

In the previous sections, we have extensively discussed the immunological characteristics of individuals with DM and TB and the immunological features of individuals with comorbid DM and TB. It has been observed that DM significantly impacts the host immune system and reduces the individual's ability to defend against MTB infection. In this section, we will continue to explore the impact of DM on the prevention, diagnosis, and treatment of TB from an immunological perspective. A comprehensive understanding of the immunological interplay between these two diseases is crucial for effective management. Strategies to address this impact should include optimizing blood glucose control, integrating TB screening, and diagnostic approaches that consider diabetes‐related immunological changes, and ensuring close collaboration among healthcare professionals involved in the management of both diabetes and TB.

### 4.1 The impact of DM on the prevention of TB

In addition to managing TB patients and preventing disease transmission, protecting vulnerable populations is crucial for TB prevention. It has been reported that approximately 23% of the global population has LTBI, and 5%–10% of individuals will progress to active tuberculosis (ATB) in their lifetime.^[^ ^134^, ^135^ ^]^ Concerningly, individuals with weakened immune systems are at higher risk of MTB infection, significantly increasing the challenges of prevention. For instance, numerous studies have indicated that DM is a major risk factor for TB, and reducing the incidence of DM has significant benefits in alleviating the burden of TB.^[^ ^136^ ^]^ Therefore, we further explore the factors contributing to increased susceptibility to TB in the presence of DM, focusing on innate and adaptive immune mechanisms as the underlying reasons. Among them, studies have suggested that poor glycemic control, IR, DM comorbidities, and genetic factors in DM patients may contribute to the increased risk of infection.

#### 4.1.1 Association between poor glycemic control and increased TB risk

DM patients often experience hyperglycemia, which impairs immune system function and makes the body more susceptible to MTB invasion. Elevated blood glucose levels can decrease the killing ability of macrophages and other immune cells against MTB, thereby increasing the risk of TB infection. Previous studies have also shown that poor glycemic control is associated with an increased risk of TB (Table 4).^[^ ^137^, ^138^, ^139^, ^140^, ^141^, ^142^, ^143^, ^144^, ^145^, ^146^, ^147^, ^148^, ^149^, ^150^ ^]^ These studies measured the blood glucose levels of patients using glycated HbA1c, fasting plasma glucose (FPG) or fasting blood sugar (FBS), and postprandial glucose levels after 2 h (2HPP). HbA1c is a substance formed by the combination of hemoglobin and glucose that accurately reflects a patient's blood glucose levels over the past 3 months, serving as a reliable indicator of glycemic control. FPG or FBS measures blood glucose levels after an 8‐h fasting period and is commonly used to screen for diabetes. Among the 14 studies reviewed, most focused on glycemic control in individuals with T2DM and found a higher number of individuals with HbA1c ≥7% or FPG/FBS > 130 mg dL^−1^ among those with both DM and TB compared to those with DM alone. Furthermore, two studies used immunological tests to screen for LTBI in DM patients. Most patients with positive results had poor glycemic control, suggesting that poor glycemic control may be a risk factor for LTBI in DM patients.^[^ ^139^, ^142^ ^]^ Similarly, a study investigating the association between type 1 DM and TB found a strong correlation between poor glycemic control and TB risk.

**TABLE 4:** Poor blood sugar control is associated with an increased risk of TB.

Ref. country, year	Research design	Simple size (*n*)	Blood sugar: *n* (incidence rate or %)	Results	Conclusion
[137] China, 2008	Prospective cohort study	42,116 (DM: 6444 NDM: 35,672)	DM‐TB: (1) HbA1c ≥ 7%, *n *= 64 (422/100,000) (2) HbA1c < 7%, *n *= 11 (136/100,000)	Subjects with HbA1c ≥ 7% had a 3.11 times risk of developing active TB (adjusted OR = 3.11, 95% CI: 1.63–5.92).	Blood sugar control was shown to be a major factor in the increased risk of TB.
[138] India, 2013	Descriptive study	7080 (DM‐TB: 47 DM‐NTB: 6113)	DM‐TB: (1) HbA1c ≥ 7%, *n *= 37 (78.7%) (2) HbA1c < 7%, *n *= 7 (14.9%)	DM‐TB patients have poor blood sugar control.	Screening of DM patients for TB was feasible in a tertiary care hospital.
[139] Mexico, 2015	Cross‐sectional study	DM: 600 [TST (+): 308 TST (‐): 292]	DM‐LTBI: (1) HbA1c > 7%, *n *= 199 (64.8%) (2) HbA1c < 7%, *n *= 109 (35.2%)	Subjects with HbA1c > 7% had a 2.52 times risk of developing LTBI (adjusted OR = 2.52, 95% CI: 1.10–8.25).	Poor glycemic control is a risk factor for LTBI in DM patients.
[140] China, 2016	Observational cohort study	122,402 (DM: 11,260 NDM: 110,782)	DM‐TB: (1) FPG > 130 mg dL^−1^, *n *= 54 (85.7%) (2) FPG ≤ 130 mg dL^−1^, *n *= 9 (14.2%)	Subjects with poorly controlled DM patients had a 2.21 times risk of developing TB (adjusted OR = 2.21, 95% CI: 1.63–2.99).	Good blood sugar control may alter the risk of TB in people with diabetes.
[141] Barcelona, 2022	Retrospective cohort study	8004 (DM: 7956 DM‐TB: 48)	DM‐TB: (1) HbA1c ≥ 7.5%, *n *= 24 (120.5/100,000) (2) HbA1c < 7.5%, *n *= 22 (70.3/100,000) (3) HbA1c ≥ 8%, *n *= 21 (143.0/100,000) (4) HbA1c < 7%, *n *= 25 (68.6/100,000) (5) HbA1c ≥ 9%, *n *= 16 (183.8/100,000) (6) HbA1c < 7%, *n *= 30 (70.6/100,000)	The risk of TB is positively associated with the degree of glycemic control. The risk of TB in patients with moderate glycemic control is 1.80 times higher (adjusted HR = 1.80, 95% CI: 0.60–5.42). The risk of TB in patients with poor glycemic control is 2.06 times higher (adjusted HR = 2.06, 95% CI: 0.67–6.32). The risk of TB in patients with very poor glycemic control is 2.82 times higher (adjusted HR = 2.82, 95% CI: 0.88–9.06).	DM subjects with worse glycemic control show a trend toward a higher risk of developing TB.
[142] Indonesia, 2022	Cross‐sectional study	T2DM: 242 [QFT (+): 82 QFT (−): 160]	DM‐LTBI: (1) HbA1c < 7%, *n *= 22 (26.8%) (2) HbA1c = 7% −9.9%, *n *= 43 (52.5%) (3) HbA1c ≥ 10%, *n *= 17 (20.5%)	Subjects with HbA1c > 7% had a 2.1 times risk of developing LTBI (adjusted OR = 2.13; 95% CI: 1.074–4.225).	A higher HbA1c level may be a predictor of LTBI in T2DM patients.
[143] South Africa, 2009	Cross‐sectional study	T1DM: 263	(1) T1DM‐NTB: HbA1c, mean = 10.6%, *n* = 233 (2) T1DM‐Prevalent TB: HbA1c mean = 12.4%, *n* = 9 (3) T1DM‐Ever TB: HbA1c mean = 13.3%, *n* = 25	Poor glycaemic control (HR = 1.39, 95% CI: 1.18–1.63 per unit increase in glycated HbA1c) was associated with prevalent TB disease.	There is a high prevalence of TB disease in diabetic children and adolescents.
[144] Kiribati, 2019	Case–control study	773 (TB: 275 NTB: 498)	(1) TB HbA1c Median = 6.0%, *n* = 275 (2) NTB HbA1 Median = 5.6%, *n* = 498	Pre‐diabetes (HbA1c 5.7%–6.4%) increased the odds of having TB by 1.5 times (OR = 1.5, 95% CI: 1.1–2.3; *p *< 0.05). Well‐controlled diabetes increased the chances of developing TB by 2.7 times (OR = 2.7, 95% CI: 1.7–4.5; *p* < 0.001), while uncontrolled diabetes increased the odds by 4.3 times (OR = 4.3, 95% CI: 2.6–7.2; *p* < 0.001).	As the HbA1c rose so did the odds of TB.
[145] India, 2017	Case–control study	451 (DM‐TB: 152 DM: 299)	DM‐TB: (1) HbA1c < 7%, *n* = 19.0 (12.2%) (2) HbA1c7%−8%, *n* = 10.0 (6.60%) (3) HbA1c > 8%, *n* = 78 (51.7%) (4) FBS < 70 mg dL^−1^, *n* = 6.0 (4.0%) (5) FBS = 70–100 mg dL^−1^, *n* = 15(9.9%) (6) FBS > 100 mg dL^−1^, *n* = 111 (87.4%) (7) Urine sugar (1+), *n* = 12 (7.9%) (8) Urine sugar (2+), *n* = 13 (8.6%) (9) Urine sugar (3+), *n* = 30 (19.9%)	HbA1c value < 7 is an associated protective factor for TB occurrence (OR = 0.52, 95% CI: 0.29–0.93).	Poor glycemic control among people with diabetes is a risk factor for TB occurrence.
[146] Brazil, 2019	Case–control study	DM‐TB: 45 DM: 90	DM‐TB: (1) HbA1c, mean = 8.81%, *n* = 45 (2) HbA1c1 before TB = 9.43%, *n* = 22 (3) FBS = 195.12 mg dL^−1^, *n* = 45 DM: (1) HbA1c, mean = 7.86%, *n* = 85 (2) HbA1c1 before TB = 7.86%, *n* = 85 (3) FBS = 145.04 mg dL^−1^, *n* = 90	FBS were associated with TB in DM (Adjusted OR = 1.017, 95% CI: 1.007–1.026).	Blood sugar control increases the chance that people with diabetes will develop tuberculosis.
[147] Korea, 2012	Case–control study	492 DM‐TB: 142 TB: 368	DM‐TB: (1) HbA1c ≥ 7%, *n* = 74 (52.1%) (2) HbA1c < 7%, *n* = 25 (17.6%)	Uncontrolled DM was a significant risk factor for a positive sputum culture at 2 months (OR = 4.316; 95% CI: 1.306–14.267; *p* = 0.017).	TB patients with uncontrolled DM should be carefully managed and treated.
[148] Kenya, 2017	Cross‐sectional study	TB: 454	TB: (1) HbA1c ≥ 6.5, *n* = 23 (5.1%) (2) HbA1c = 5.7–6.4, *n* = 170 (37.5%) (3) HbA1c < 5.7, *n* = 260 (57.45%)	The odds of having pre‐DM or DM among TB patients were 42.6% (95% CI: 38.0–47.3).	High rates of pre‐diabetes and DM were found in adult TB patients.
[149] Thailand, 2022	Prospective cohort study	DM‐TB: 92 NDM‐TB: 124	DM‐TB: (1) FPG median = 153 mg dL^−1^, *n* = 92 (2) HbA1c median = 7.0%, *n* = 92 NDM‐TB: (1) FPG median = 100 mg dL^−1^, *n* = 124 (2) HbA1c median = 5.4 mg dL^−1^, *n* = 124	Having pre‐existing underlying impaired fasting glucose (OR = 8.03, *p *< 0.001) was associated with newly diagnosed DM cases among patients with TB.	The prevalence of DM in patients with TB in Thailand was unexpectedly high.
[150] Ghana, 2018	Cross‐sectional study	TB: 146	TB: (1) FPG = 6.1–7 mmol L^−1^, *n* = 8 (5.48%) (2) FPG > 7.1 mmol L^−1^, *n* = 5 (3.42%) (3) 2HPP = 6.1–7 mmol L^−1^, *n* = 42 (28.77%) (4) 2HPP > 7.1 mmol L^−1^, *n* = 17 (11.64%)	In TB patients, the prevalence of abnormal fasting blood glucose was 8.9% (95% CI: 5.21‐14.82%), and the prevalence of abnormal postmeal blood glucose was 40.4% (95% CI: 32.68‐48.65%).	The prevalence of dysglycaemia was high among smear‐positive TB patients in Ghana.

Abbreviations: DM, diabetes mellitus; FBS, fasting blood sugar; FPG, fasting plasma glucose; HbA1c, glycated hemoglobin A1c; 2HPP, 2‐hour postprandial values; IGRA, interferon‐gamma release assay; LTBI, latent tuberculosis infection; QFT, QuantiFERON‐TB test; TB, tuberculosis; TST, tuberculin skin test.

Moreover, Lee et al., in a 5‐year follow‐up study of 123,546 participants, examined the association between glycemic control and the incidence of TB. They found that patients with poor glycemic control (FPG > 130 mg dL^−1^) had double the risk of developing TB compared to patients without DM (NDM) and well‐controlled DM patients (FBG ≤ 130 mg dL^−1^) (HR 2.21, 95% CI 1.63–2.99, *p* < 0.001).^[^ ^140^ ^]^ Similarly, Wang et al. conducted a case–control study involving 315 patients and also found an association between poor glycemic control and increased TB risk.^[^ ^151^ ^]^ Furthermore, a prospective study involving 4690 DM patients and evaluating their blood glucose control using HbA1c revealed that patients with HbA1c > 7% had approximately three times the risk of developing ATB compared to patients with HbA1c < 7% (HR 3.11, 95% CI 1.63–5.92).^[^ ^137^ ^]^ Moreover, as mentioned earlier, in the context of hyperglycemia, DM patients experience a decrease in the frequency and antimicrobial function of innate immune cells such as macrophages, dendritic cells, and NK cells, as well as delayed activation of adaptive immune cells. These immune suppressions weaken MTB's ability to resist, thereby increasing the risk of TB. In summary, these studies demonstrate that poor glycemic control significantly increases the risk of MTB infection. However, there is currently limited research on the exact role of clinical diabetes control in reducing the risk of infection. A modeling study that covered 13 countries predicted that continuing to reduce the incidence of DM until 2025 could prevent 7.8 million new TB cases and 1.5 million TB‐related deaths by 2035.^[^ ^136^ ^]^ Consequently, blood glucose management for DM patients may hold significant implications for reducing the occurrence of TB.

#### 4.1.2 IR associated with increased TB risk

IR is a key feature of T2DM, and current research suggests that IR may increase susceptibility to TB through its interaction with ROS and vitamin D.^[^ ^152^, ^153^ ^]^ Chao et al. found that elevated resistin levels in the serum of IR patients impaired ROS production, thus reducing the bactericidal function of macrophages in individuals with DM‐TB comorbidity.^[^ ^152^ ^]^ Resistin, a 12 kDa soluble serum protein, plays a crucial role in macrophage bactericidal activity, while ROS serves as an important mechanism in this process. Increased resistin levels can promote IR while inhibiting ROS production by immune cells. Chao et al. also observed significantly elevated resistin levels in both DM and severe TB patients.^[^ ^152^ ^]^ This suggests that the elevated resistin levels in IR patients may contribute to reduced bactericidal activity of macrophages, thereby increasing susceptibility to TB or exacerbating the disease.^[^ ^152^ ^]^ Additionally, vitamin D plays a pivotal role in the pathogenesis of TB and immune system modulation, particularly in influencing macrophage antimicrobial activity, autophagy, and phagolysosome fusion.^[^ ^153^ ^]^ Research by Yong Chen et al. identified a negative correlation between serum 25(OH)D levels and homeostatic model assessment of IR (HOMA‐IR) in LTBI.^[^ ^153^ ^]^ Consequently, reduced vitamin D levels in IR patients may further compromise the ability to clear MTB and increase the risk of infection. Moreover, another study has shown that IR levels have an impact on the radiologic features of DM‐TB patients.^[^ ^154^ ^]^ Specifically, elevated IR levels were strongly associated with pulmonary lesions, bronchial distension, thin‐walled cavities, and bronchial abnormalities observed on CT scans.^[^ ^154^ ^]^ These findings suggest that IR levels can effectively reflect the severity of TB disease. In summary, there is a close relationship between IR and the risk of TB infection, which may contribute to the clinical aggravation of TB in affected individuals.

#### 4.1.3 Association between DM complications and increased TB risk

Many individuals with DM may experience various complications, and research suggests a correlation between DM complications and the risk of TB. A prospective study found a positive association between the number of DM complications and TB risk, with DM patients having three or more complications being at more than a twofold increased risk for TB.^[^ ^155^ ^]^ Furthermore, smoking, vitamin D deficiency, and lipid abnormalities have been found to not only contribute to DM‐related renal and vascular complications but may also be associated with the reactivation of LTBI.^[^ ^156^ ^]^ Lesnic et al. further demonstrated that the presence of different types of complications in individuals with DM increases the risk of developing TB, possibly due to long‐term complications leading to decreased immune responses.^[^ ^157^ ^]^

#### 4.1.4 Possible genetic factors in DM‐TB pathogenesis

It is well known that Asian countries such as India and China have a high burden of both DM and TB. Walker et al. investigated 3461 TB patients in England and attributed 384 cases (11.1%) to DM. The proportion of DM‐TB association among Asians was 55%, while it was 22% for Caucasians and 23% for Blacks, suggesting a racial specificity in the occurrence of these diseases, with Asians having a higher incidence of DM and TB.^[^ ^158^ ^]^ Similarly, Suwanpimolkul et al. recruited 791 TB patients, including individuals from the United States (29.2%), Asia (26.7%), Mexico (11%), and other countries (33.1%). They found a prevalence of DM among TB patients at 15.9%, with the highest impact among the non‐US population affected by both diseases.^[^ ^159^ ^]^

These findings suggest that susceptibility factors between DM and TB may have geographic variations and could be influenced by racial/genetic differences. Park et al. discovered a strong genetic association between the functional polymorphism of aldehyde dehydrogenase 2 (ALDH2 Glu487Lys) and the risk of TB, with a higher frequency observed in Asian populations, indicating that TB might have been a localized disease.^[^ ^160^ ^]^ Similarly, Radha et al. found that the peroxisome proliferator‐activated receptor (PPAR)‐γ Pro12Ala polymorphism had a protective effect against DM in Caucasians but not in South Asians.^[^ ^161^ ^]^ This suggests that genetic characteristics in Asian populations may make them more susceptible to the impacts of DM and TB. Additionally, some studies have identified associations between susceptibility to DM‐TB and host genetic variations. Zhong et al. extracted genetic data from 3,421,574 participants in the UK Biobank and found that TB‐associated variations in the HLA‐DRA‐DQA1 region (rs2894257) were associated with both T1DM and T2DM. Another variation (rs3135359) in the same region was associated with type 1 DM, while a third variation (rs4733781) was moderately associated with T2DM.^[^ ^162^ ^]^ Therefore, an in‐depth understanding of these genetic factors could facilitate a more comprehensive investigation of the specific genetic risk factors contributing to the complex interplay between DM and TB, shedding light on their combined pathogenesis and potential implications for clinical management.

### 4.2 The impact of DM on the immunodiagnosis of TB

The diagnosis of TB usually requires multiple diagnostic tests, including clinical manifestations, pathogen identification, imaging, molecular biology, and immunological examinations.^[^ ^163^ ^]^ First, sputum culture is considered the “gold standard” for diagnosing active TB, but the results take several weeks and have a low positive detection rate, which may lead to delays in diagnosis and treatment.^[^ ^164^ ^]^ Second, imaging techniques can non‐invasively and rapidly detect infected lesions but cannot provide etiological diagnosis.^[^ ^163^ ^]^ Last, although molecular biology methods have higher sensitivity and specificity, they are costly and technically demanding, which increases the economic burden on patients and limits the widespread use of this technology in TB diagnosis, especially in resource‐limited areas or developing countries. Fortunately, immunological examinations have advantages such as early detection, rapidity, sensitivity, and convenience of specimen collection, and have become important auxiliary diagnostic criteria for TB.^[^ ^165^ ^]^

TST involves the injection of PPD of tuberculin into the skin to observe delayed hypersensitivity reactions. New TST methods have emerged, such as C‐TB, Diaskintest, and EC skin tests. However, TST has a lower specificity and is susceptible to cross‐reactions from BCG and non‐tuberculous mycobacteria (NTM). On the other hand, IGRA performs TB diagnosis by detecting the release of IFN‐γ after specific antigen stimulation, demonstrating high specificity, and it is not affected by BCG and NTM. Currently, there are various IGRA methods available, such as AdvanSure^™^ TB‐IGRA ELISA, Wantai TB‐IGRA, Standard E TB‐Feron (TBF), QIAreach QFT, ichroma^™^ IGRA‐TB, VIDAS TB‐IGRA, and T‐Track TB. Although both TST and IGRAs are based on the body's immune response, their sensitivity may be affected in patients with compromised immunity, such as those with DM (Table 5).^[^ ^166^, ^167^, ^168^, ^169^, ^170^ ^]^ For instance, Yijun He et al. evaluated the prevalence of LTBI in patients with T2DM and performed TST, C‐TST, and IGRA tests in 404 patients aged 57 years (53–60 years for IQR), with results of 9.65% (39/404), 10.40% (42/404), and 14.85% (60/404), respectively.^[^ ^166^ ^]^ Although the IGRA showed the highest positivity rate in this study, it was still significantly lower than that of the general population (age: 59 years, IQR: 54–64) in the same study center.^[^ ^166^, ^171^ ^]^ Specifically, this study showed an IGRA positivity rate of 20.79% (4259/20486) in the general population, which was significantly higher than in T2DM patients.^[^ ^166^, ^171^ ^]^ However, previous studies have indicated that the risk of TB is higher in DM patients than in the general population, which contradicts the aforementioned findings and may suggest a decreased sensitivity of immunological examinations in DM patients. Similarly, Santos et al. retested TST and IGRA in 41 confirmed cases of DM‐TB patients, with results showing an IGRA positivity rate of 78% (32/41 cases), TST‐5 mm positivity rate of 76% (31/41 cases), and TST‐10 mm positivity rate of 66% (27/41 cases).^[^ ^167^ ^]^ Santos et al. observed false negatives in confirmed patients, suggesting that DM may decrease the sensitivity of TST and IGRA.^[^ ^167^ ^]^ Furthermore, Chee et al. compared the sensitivity of two commercial IGRAs for IGRA. They found that the decreased performance of IGRA was not independently associated with DM. Still, a large proportion of TB patients with uncertain or failed results for QFT‐IT and T‐SPOT were often concomitant with DM.^[^ ^172^ ^]^ Meanwhile, Nantha et al. also found a certain level of immunosuppression in DM patients.^[^ ^173^ ^]^ Therefore, a lower TST threshold may be required when detecting MTB infection in DM patients.

**TABLE 5:** Effect of DM on immunological diagnosis of TB.

Ref., country, year	Research design	Sample size (*n*)	Immunological examination: positive in DM patient *n* (%)	Result	Conclusion
[166] China, 2022	Prospective study	DM: 404	(1) TST, *n* = 39 (9.65%) (2) C‐TST, *n* = 42 (10.40%) (3) IGRA, *n* = 60 (14.85%)	The concordance of TST and C‐TST results with IGRA results was 86.39% (349/404) and 92.08% (372/404) with a Kappa coefficient of 0.37 (95% CI 0.24–0.50) and 0.64 (95% CI: 0.53–0.76), respectively.	In patients with T2DM, C‐TST showed higher consistency with IGRA than TST.
[167] Portugal, 2022	Retrospective cohort study	TB: 727 DM‐TB: 41	(1) TST‐5 mm, *n* = 31 (75.6%) (2) TST‐10 mm, *n* = 27 (65.8%) (3) IGRA, *n* = 32 (78.0%)	IGRA and TST‐5 mm show agreement (kappa, 0.795) (*p *< 0.001). IGRA and TST‐10 mm show agreement (kappa, 0.585) (*p *< 0.001).	Comorbidities can lower the sensitivity of individual tests. Combining both tests could enhance the detection of infection cases compared to using either test alone.
[168] China, 2010	Prospective cohort study	DM: 84	ELISPOT, *n* = 54	The sensitivity of ELISPOT in poorly controlled blood glucose is 77.4% (24/31). The sensitivity of ELISPOT in poorly controlled blood glucose is 90.1% (10/11).	The negative predictive value of the ELISPOT assay was significantly higher in patients with adequate glycaemic control (90% vs. 56.3%).
[169] America, 2015	Retrospective study	TB: 300 DM‐TB: 44	TST, *n* = 41 (93.2%) QFT, *n* = 26 (59.1%)	TST sensitivity in DM‐TB patients:93.2% (95% CI: 81.8–97.7). QFT sensitivity in DM‐TBpatients:59.1% (95% CI: 44.4–72.3).	QFT sensitivity was lower than that of TST, especially in patients with DM.
[170] Tanzanian, 2014	Cross‐sectional study	TB: 187	DM: Normal glucose tolerance: 126 (67.4%) Pre‐diabetes: 45 (24.1%) Diabetes: 16 (8.6%) QFT result: Negative: 25 (13.4%) Positive: 134 (71.7%) Indeterminate: 28 (15.0%)	The negative test result was more common among TB patients with pre‐diabetes (OR = 3.1, 95% CI: 1.2–8.2, *p* = 0.022). The increase of FBG was negatively correlated with IFN‐γ (B‐0.3, 95% CI 0.6–0.03, *p *= 0.033).	In patients with diabetes, the effectiveness of IFN‐γ in detecting LTBI may be questionable.

Abbreviations: C‐TST, creation tuberculin skin test; DM, diabetes mellitus; IGRA, interferon‐gamma (IFN‐γ) release assay; LTBI, latent tuberculosis infection; QFT, QuantiFERON‐TB test; TB, tuberculosis; TST, tuberculin skin test.

It is worth noting that IGRA assists in diagnosing TB by detecting the levels of IFN‐γ. Previous studies have found a decrease in the secretion of IFN‐γ in DM patients, especially in those with poor blood glucose control,^[^ ^24^ ^]^ which may affect the sensitivity of detecting TB in DM patients. Moreover, in a mouse model, it was found that DM‐TB mice exhibited lower levels of IFN‐γ secretion, particularly under high blood glucose conditions.^[^ ^101^ ^]^ These findings suggest a close correlation between the level of glucose control and the secretion level of IFN‐γ. In addition, TAN et al. studied the sensitivity of ELISPOT in DM‐TB patients and found that it reached 90.1% in patients with good blood glucose control but only 77.4% in patients with poor blood glucose control.^[^ ^168^ ^]^ This indicates that immunosuppression in DM patients with poor blood glucose control can significantly decrease the levels of IFN‐γ, leading to decreased sensitivity in IGRA.

### 4.3 Impact of DM on the treatment of TB

DM can affect the efficacy and prognosis of TB treatment. Hyperglycemia associated with DM may impair the immune response and alter drug metabolism, affecting anti‐TB drug efficacy. This may lead to prolonged treatment duration, higher rates of treatment failure, and an increased risk of DR‐TB. In addition, managing comorbidities such as diabetic complications during TB treatment can be challenging and requires careful coordination and monitoring by healthcare providers. This section will explore the impact of DM on the treatment of TB patients to provide new ideas and insights for early and accurate treatment of DM‐TB patients.

#### 4.3.1 Impact of DM on the clinical presentation of TB patients

Previous studies have shown that compared to pure TB, DM‐TB patients have more severe clinical manifestations, which increases the difficulty of treatment.^[^ ^174^ ^]^ It has been found that a large proportion of DM‐TB patients exhibit cavitation, infiltration, fibrosis, or multiple lesions, indicating worse radiological findings in TB patients with DM.^[^ ^174^ ^]^ Furthermore, some studies have found that DM‐TB patients are more likely to have lower lung field lesions and cavitation compared to TB patients without DM,^[^ ^147^, ^175^, ^176^, ^177^, ^178^, ^179^, ^180^ ^]^ and it is significantly correlated with blood glucose control.

#### 4.3.2 Impact of DM on DR‐TB

DR‐TB is one of the important challenges in global public health. It is insensitive to conventional first‐line anti‐TB drugs and usually requires long‐term treatment with more side effects from second‐line and third‐line anti‐TB drugs. This increases the economic burden on patients and lowers the cure rate. Previous studies have shown that DM may increase the risk of DR‐TB.^[^ ^181^ ^]^ A meta‐analysis covering 15 countries found a significant association between DM and DR‐TB,^[^ ^181^ ^]^ and other studies have also found that DM increases the risk of DR‐TB by 2 to more than 8 times.^[^ ^175^, ^182^, ^183^, ^184^, ^185^, ^186^ ^]^ Currently, some possible explanations have been proposed for the increased risk of DR‐TB in DM. First, the lack of effective drug concentration in DM patients may be related to drug resistance. Studies have found that DM patients have reduced clearance ability for isoniazid and rifampicin, leading to decreased killing effect on MTB, and DM patients show a slow response to TB treatment.^[^ ^187^ ^]^ In addition, studies have also found that DM‐TB patients have lower blood concentrations of rifampicin compared to TB patients without DM, making DM an independent risk factor for decreased rifampicin concentration.^[^ ^188^, ^189^ ^]^ Second, some studies have proposed that therapeutic agents for TB can cause the worsening of blood glucose in DM patients, such as rifampicin and rifapentine.^[^ ^190^ ^]^ For instance, a study by Zheng et al. found that rifampicin reduced the efficacy of glibenclamide through induction of p‐glycoprotein and CYP3A4.^[^ ^191^ ^]^ Specifically, their study showed that patients who took multiple oral doses of rifampicin had 63% and 48% lower AUC and C_max_ of glibenclamide and higher blood glucose levels compared to controls.^[^ ^191^ ^]^ Unlike rifapentine, which does not induce p‐glycoprotein, it reduces serum concentrations of rosiglitazone and gliclazide by inducing CYP2C8.^[^ ^190^ ^]^ Unexpectedly, rifapentine has also been shown to cause elevated blood glucose in non‐diabetic patients.^[^ ^192^ ^]^ These suggest that therapeutic drugs for TB can directly or indirectly lead to the deterioration of glycemic control.^[^ ^190^ ^]^ Thus, the vicious cycle of TB and diabetes interacting may lead to consequences similar to those of drug abuse, one of the most common causes of drug resistance.^[^ ^192^, ^193^, ^194^ ^]^ Last, bacterial genetics may be related to DR‐TB. Yuan et al. found that drug‐resistant strains have genetic diversity and lower clustering rates, and DM is considered the most common comorbidity in these DR‐TB cases.^[^ ^195^ ^]^ Additionally, Gagneux et al. found that isoniazid‐resistant strains are associated with KatG S315T or inhA promoter mutations, and impaired ROS production in T2DM patients may make strains with katG gene mutations more likely to survive.^[^ ^182^, ^196^ ^]^

#### 4.3.3 Impact of DM on the treatment outcomes of TB patients

Previous studies have also found an association between DM and poor treatment outcomes in TB (Table 6). An analysis of 14 studies included in Table 6 showed that DM‐TB patients have a higher risk of treatment failure and death.^[^ ^197^, ^198^, ^199^, ^200^, ^201^, ^202^, ^203^, ^204^, ^205^, ^206^, ^207^, ^208^, ^209^, ^210^ ^]^ Among them, the highest odds ratio (OR) for treatment failure was OR: 9.80 (0.32–30.07), and the lowest OR was OR: 1.176 with a 95% CI of 0.310–4.457. This indicates a significant impact of DM on the treatment outcomes of TB, and DM may be an independent risk factor for TB treatment failure.

**TABLE 6:** The impact of DM on treatment outcomes in TB patients.

Ref. Country, year	Research design	Sample size (*n*)	Treatment regimen	Treatment outcomes *n* (%)	Results	Conclusion
[197] China, 2017	Prospective study	TB: 21,414 DM‐TB: 3331	WHO recommended HRZ‐containing standard regimens	(1) Treatment success: NDM‐TB, *n* = 13,726 (78.5%) DM‐TB, *n* = 2368 (73.9%) (2) Still on treatment: NDM‐TB, *n* = 1340 (7.7%) DM‐TB, *n* = 368 (11.5%) (3) Death: NDM‐TB, *n* = 1152 (6.6%) DM‐TB, *n* = 316 (9.9%)	Patients with DM‐TB complications have a lower success rate than patients with TB (adjusted OR = 0.83, 95% CI: 0.75–0.93, *p* = 0.001).	DM adversely affected the clinical presentation and treatment response of TB, but there was no difference in the drug resistance and relapse rates.
[198] Pakistan, 2016	Prospective cohort study	TB: 614 DM‐TB: 113	NTP, WHO	(1) Treatment success, *n* = 434 (86%) (2) Treatment failed, *n* = 69 (14%)	Patients with DM‐TB were more likely to have adverse outcomes than NDM‐TB (OR = 2.6, 95% CI: 1.48–4.56, *p* = 0.001).	Patients with DM‐TB were more likely to have adverse outcomes.
[199] South Korea, 2017	Retrospective	TB: 1044 DM‐TB: 253	WHO guidelines	(1) Treatment success: NDM‐TB, *n* = 649 (84.9%) DM‐TB, *n* = 177 (74.4%) (2) Unsuccessfully treated: NDM‐TB, *n* = 115 (15.1%) DM‐TB, *n* = 61 (25.6%) (3) Died: NDM‐TB, *n *= 28 (3.7%) DM‐TB, *n* = 7 (2.9%)	Diabetes was a risk factor for unsuccessful treatment outcomes OR = 1.67, 95% CI (1.03–2.70), *p* = 0.039.	Diabetes may delay sputum conversion and adversely affect treatment outcomes.
[200] China, 2015	Retrospective	TB: 1473 DM‐TB: 705	WHO guidelines	(1) Treatment success: DM‐TB, *n* = 590 (83.7%) NDM‐TB, *n* = 687 (89.5%) (2) Treatment failed: DM‐TB, *n* = 22 (3.1%) NDM‐TB, *n* = 17 (2.2%) (3) Died: DM‐TB, *n* = 79 (11.2%) NDM‐TB, *n* = 61 (7.9%)	DM‐TB patients were significantly less likely to have treatment success (OR = 0.61, 95% CI: 0.45–0.82). DM‐TB patients were significantly increased risk of death (OR = 1.46, 95% CI: 1.03–2.08).	Poor glycemic control is associated with poor TB treatment outcome, and improved glycemic control may reduce the influence of diabetes on TB.
[201] Maryland, 2009	Retrospective cohort study	TB: 297 DM‐TB: 42	DOTS	Died: DM‐TB, *n* = 6 (14.3%) NDM‐TB, *n *= 20 (7.8%)	Mortality of tuberculosis patients affected by diabetes (adjusted OR = 6.5, 95% CI: 1.1–38.0, *p* = 0.039).	DM was a risk factor for death in Maryland TB patients.
[202] Armenia, 2020	Retrospective cohort	TB: 621 DM‐TB: 36	WHO guidelines	(1) Treatment success: DM‐TB, *n* = 26 (72.22%) NDM‐TB, *n* = 484 (82.74%) (2) Treatment failed: DM‐TB, *n* = 4 (11.11%) NDM‐TB, *n* = 34 (5.81%) (3) Died: DM‐TB, *n *= 3 (8.33%) NDM‐TB, *n* = 34 (5.81%)	The odds ratio for treatment failure among DM‐TB patients was 8.99 (95% CI: 2.591–32.23), *p *< 0.001.	Diabetes comorbidity had a negative effect on TB treatment outcomes.
[203] Japan, 2014	Retrospective	TB: 260 DM‐TB: 69	Japanese guidelines: DOTS	Cavity: DM‐TB, *n* = 49 (71%) NDM‐TB, *n* = 87 (45.6%)	DM is the major determinant for delayed sputum culture conversion at 2 months after treatment, with OR = 3.108 (95% CI: 1.698–5.708), *p* = 0.0003.	Compared with non‐DM patients, patients with DM had an increase in pulmonary cavities and a significantly longer transformation time of sputum culture.
[204] Mexico, 2012	Prospective	TB: 1262 DM‐TB: 374	Mexico's National TB Control Program guidelines; WHO; DOTS.	(1) Treatment success: DM‐TB, *n* = 259 (71.35%) NDM‐TB, *n* = 605 (71.43%) (2) Treatment failed: DM‐TB, *n* = 17 (4.68%) NDM‐TB, *n* = 19 (2.24%) (3) Died: DM‐TB, *n* = 11 (3.03%) NDM‐TB, *n *= 36 (4.25%)	Patients with DM‐TB had a higher probability of treatment failure (adjusted OR = 2.93, 95% CI: 1.18–7.23), recurrence (adjusted HR = 1.76, 95% CI 1.11–2.79), and relapse (adjusted HR = 1.83, 95% CI: 1.04–3.23).	DM‐TB patients exhibit severe clinical manifestations, delayed sputum conversion, and a higher likelihood of treatment failure, recurrence, and relapse.
[205] India, 2016	Prospective	TB: 316 DM‐TB: 50	RNTCP	(1) Treatment success: DM‐TB, *n* = 26 (72.2%) NDM‐TB, *n* = 93 (62%) (2) Treatment failed: DM‐TB, *n* = 2 (5.6%) NDM‐TB, *n* = 3 (2.0%) (3) Died: DM‐TB, *n *= 1 (2.8%) NDM‐TB, *n* = 9 (6.0%)	The risk of unsuccessful treatment outcomes in DM‐TB patients was OR = 1.176, 95% CI (0.310–4.457).	DM affects treatment outcomes for TB patients.
[206] Ethiopia, 2016	Prospective cohort study	TB: 1314 DM‐TB: 109	WHO guidelines, DOTS	(1) Treatment success: DM‐TB, *n* = 26 (23.9%) NDM‐TB, *n* = 291 (24.1%) (2) Treatment failed: DM‐TB, *n* = 1 (0.9%) NDM‐TB, *n* = 14 (1.2%) (3) Died: DM‐TB, *n* = 15 (13.8%) NDM‐TB, *n* = 42 (3.5%)	DM‐TB is associated with increased mortality adjusted HR = 3.96, 95% CI (1.76–8.89), *p* < 0.001.	DM is associated with increased death during TB treatment.
[207] Mexico, 2015	Retrospective	TB: 181,378 DM‐TB: 34,988	WHO, DOTS	(1) Treatment success: DM‐TB, *n* = 25,623 (86.76%) NDM‐TB, *n* = 84.302 (80.54%) (2) Treatment failed: DM‐TB, *n* = 621 (2.1%) NDM‐TB, *n *= 1666 (1.59%) (3) Died: DM‐TB, *n* = 1973 (6.68%) NDM‐TB, *n* = 10,465 (9.16%)	Treatment failure in patients with DM‐TB comorbidity adjusted OR = 1.34, 95% CI (1.11–1.61), *p *= 0.002.	Patients with DM‐TB are more likely to fail treatment.
[208] China, 2015	Prospective	TB: 1126 DM‐TB: 182	Chinese guidelines	(1) Treatment success: DM‐TB, *n* = 162 (89.0%) NDM‐TB, *n* = 904 (95.8%) (2) Treatment failed: DM‐TB, *n* = 6 (3.3%) NDM‐TB, *n* = 5 (0.5%)	Treatment failure in patients with DM‐TB comorbidity OR = 6.696, 95% CI (2.019–22.200), *p* = 0.002.	DM is associated with TB treatment failure.
[209] South Korea, 2017	Prospective	TB:661 DM‐TB: 157 (Uncontrolled‐DM: 108, Controlled‐DM: 49)	WHO guidelines	(1) Treatment success: Uncontrolled‐DM, *n* = 45 (48.4%) Controlled‐DM, *n* = 25 (62.5%) NDM‐TB, *n* = 241 (53.3%) (2) Treatment failed: Uncontrolled‐DM, *n* = 5 (5.4%) Controlled‐DM, *n* = 1 (2.5%) NDM‐TB, *n* = 3 (0.7%) (3) Died: Uncontrolled‐DM, *n* = 4 (4.3%) Controlled‐DM: NDM‐TB, *n* = 2 (0.4%)	Uncontrolled DM is an independent risk factor for unsuccessful treatment outcomes in PTB. OR = 9.80, 95% CI (0.32–30.07), *p* < 0.001.	Uncontrolled diabetes is an independent risk factor for poor treatment response in PTB.
[210] China, 2013	Retrospective cohort	TB: 1589 DM‐TB: 189	WHO guidelines	(1) Treatment success: DM‐TB, *n* = 77 (79.4%) NDM‐TB, *n *= 416 (86.1%) (2) Treatment failed: DM‐TB, *n *= 10 (10.3%) NDM‐TB, *n *= 11 (2.3%) (3) Died: DM‐TB, *n* = 2 (2.1%) NDM‐TB, *n* = 2 (0.4%)	Treatment failure in patients with DM‐TB comorbidity OR = 4.46, 95% CI (1.96–10.18), *p* < 0.001.	DM is associated with TB treatment failure.

Abbreviations: DM, diabetes mellitus; DOTS, Directly Observed Treatment Short course; H, isoniazid; Z, pyrazinamide; NDM, non‐diabetes mellitus; NTP, National Tuberculosis Control Program; PTB, pulmonary tuberculosis; R, rifampicin; RNTCP, Revised National TB Control Program; TB, Tuberculosis; WHO, The World Health Organization.

Some studies suggest that poor blood glucose control may be associated with poor treatment outcomes in TB,^[^ ^147^, ^200^, ^209^, ^211^ ^]^ and poor blood glucose control is considered one of the main reasons for poor prognosis in DM‐TB patients.^[^ ^13^ ^]^ For example, Mahishale et al. found that patients with HbA1c levels above 7% have significantly increased rates of smear non‐conversion at 2 months of treatment, as well as higher rates of treatment failure and relapse.^[^ ^211^ ^]^ Additionally, obese DM patients have chronic low‐grade inflammation, and the bacterial burden increases after TB co‐infection, leading to aggravated tissue damage, which in turn affects the clinical presentation and treatment outcomes of patients.^[^ ^93^, ^94^ ^]^ Furthermore, changes in drug metabolism dynamics caused by DM may also be a contributing factor.^[^ ^189^, ^212^ ^]^

## 5 CHALLENGES AND PROSPECTS

**FIGURE 4:**
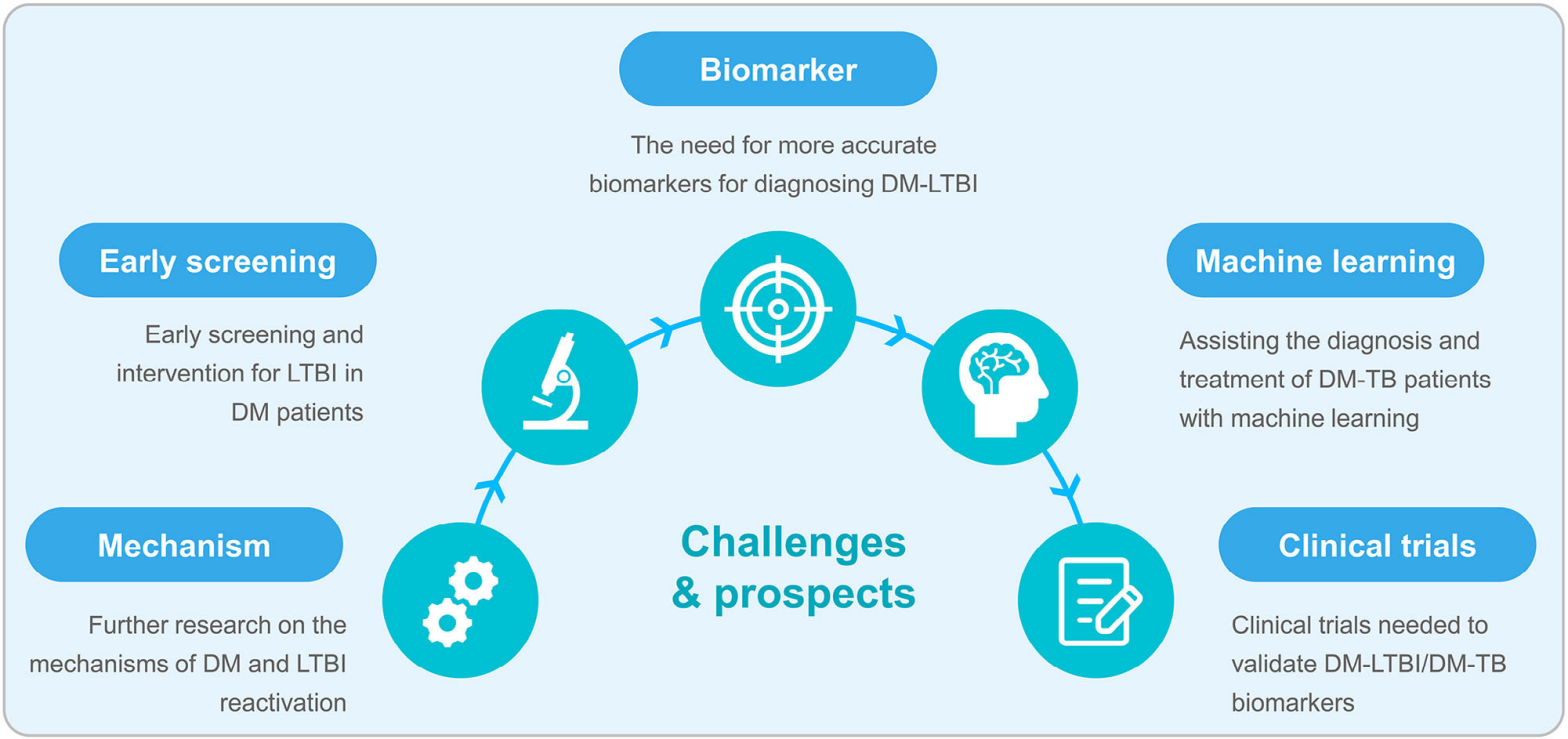
Challenges and future directions for DM‐TB control. First, further research is needed to understand the mechanisms linking DM and LTBI reactivation, potentially providing immunological targets for preventing TB in DM patients. Early screening and intervention for LTBI in DM patients is proposed, utilizing TST and IGRA for screening and implementing TPT treatment as early intervention. Additionally, since DM reduces the sensitivity of immunological tests for TB, exploring more accurate biomarkers through methods like genomics is suggested for diagnosing DM‐LTBI. Machine learning can also aid in screening and prognostic evaluation for DM‐TB patients. Finally, candidate biomarkers require clinical trials to validate their clinical utility. DM, diabetes mellitus; IGRA, interferon‐gamma release assay; TB, tuberculosis; TPT, TB preventive therapy; TST, tuberculin skin test; LTBI, latent tuberculosis infection.

With the development of the economy, the global incidence of DM is increasing year by year. An increasing number of studies have shown that DM is a major risk factor for TB, which may indirectly contribute to the increased incidence of TB. The chronic hyperglycemic state in DM patients impairs the body's normal immune response, leading to weakened immunity and increased susceptibility to TB or reactivation of LTBI. When DM is combined with TB, the interaction between the two diseases exacerbates the condition, leading to increased rates of relapse and mortality in patients. As the at‐risk populations for these two diseases increasingly overlap, they pose a serious threat to global health and have become an important issue that researchers worldwide urgently need to address. However, many unanswered questions remain about the relationships and mechanisms between DM and TB, which pose challenges for research and clinical practice. This section will discuss the challenges and future perspectives in DM‐TB control (Figure 4).

### 5.1 Further research on the mechanisms of DM and LTBI reactivation is needed

This review discusses the impact of DM on the immune mechanisms of TB patients, providing valuable information for clinical diagnosis and treatment. However, the focus on preventing TB infection in DM patients should be placed on the population with LTBI. Previous studies have shown that the occurrence of LTBI involves MTB hiding in granulomas composed of macrophages and other immune cells, maintaining a non‐disease state. However, DM leads to the activation of inflammatory cells in the body, disrupting the granulomas in LTBI patients, thus releasing and causing the hosts to develop TB disease.^[^ ^213^ ^]^ Unfortunately, the specific mechanisms of DM and LTBI reactivation remain unclear and require further in‐depth research. Understanding these mechanisms may provide immunological targets for preventing TB in DM patients.

### 5.2 Early screening and intervention for LTBI in DM patients

To prevent the reactivation of LTBI in DM patients, in addition to understanding the activation mechanisms, early detection and intervention for LTBI in DM patients are necessary to prevent its progression to ATB. Currently, diagnostic methods for detecting LTBI include TST and IGRAs. TST is widely used due to its low cost and ease of operation, while IGRA has higher sensitivity and specificity than TST and is not affected by BCG vaccination. DM patients can be screened for LTBI using either of these methods. For DM patients with coexisting LTBI (DM‐LTBI), early intervention measures can be taken to prevent disease development. First, strict blood glucose control is required for DM patients, as previous studies have indicated a link between hyperglycemia and LTBI reactivation, and good blood glucose control may significantly reduce the risk of DM reactivating LTBI.^[^ ^69^ ^]^ In addition, research has shown that LTBI patients receiving tuberculosis preventive therapy (TPT) can reduce the risk of disease occurrence by 90%. Therefore, TPT for DM‐LTBI patients may significantly lower the risk of disease. Although the WHO has not yet recommended systematic LTBI screening and TPT for DM patients, it is worth considering, given the increased incidence of DM‐TB.

### 5.3 The need for more accurate biomarkers for diagnosing DM‐LTBI

Since LTBI patients lack clinical symptoms and signs and cannot undergo auxiliary examinations such as imaging and pathogen identification, the diagnosis of LTBI relies solely on TST and IGRA. However, although TST and IGRA can indicate disease status, they cannot distinguish between LTBI and active TB, and DM may reduce the sensitivity of TST and IGRA. Therefore, more accurate means of examination are needed to screen for LTBI in DM patients accurately and prevent missed diagnoses.

This makes omics (including genomics, proteomics, metabolomics, transcriptomics, lipidomics, and immunomics) a current research hotspot. Omics is a discipline that studies the genes, proteins, and molecular interactions of organisms from a holistic perspective, discovering candidate biomarkers for DM‐LTBI using high‐throughput screening methods. Biomarkers are biochemical indicators that can mark changes in human systems, organs, tissues, cells, and their functions. Biomarkers can not only reveal molecular‐level mechanisms of disease development but also accurately assess early and low‐level damage, and provide early warning, prognostic evaluation, and a basis for precise staging of diseases.^[^ ^214^ ^]^ Relevant studies have identified biomarkers associated with the diagnosis of DM‐TB, such as the HLA‐DRA‐DQA1 region rs2894257, rs3135359, and rs4733781 in genomics.^[^ ^162^ ^]^ However, there is a lack of research on biomarkers specific to DM‐LTBI. Filling this gap may provide a more convenient, accurate, and efficient method for screening LTBI in DM patients, thereby controlling the risk of TB infection in DM patients. Additionally, utilizing machine learning for biomarker screening in omics databases can greatly improve efficiency.

### 5.4 Assisting the diagnosis and treatment of DM‐TB patients with machine learning

Machine learning is an important branch of artificial intelligence that enables automatic learning and improvement of models through data and algorithms for prediction and decision‐making with unknown data.^[^ ^215^ ^]^ The core idea of machine learning is to train models using data so that the models can predict and classify new data. Machine learning has been applied in multiple fields and can provide faster clinical diagnosis, precise treatment plans, and monitoring of disease progression in the medical field.^[^ ^216^, ^217^, ^218^, ^219^, ^220^ ^]^ For instance, Fang Xing et al. used decision trees and random forest algorithms to discover important factors influencing hypertension outcomes, and through app‐assisted self‐care, they improved chronic disease management and effectively controlled patients' blood pressure.^[^ ^221^ ^]^ Therefore, machine learning can also be used for data analysis to screen for DM‐LTBI/DM‐TB biomarkers in patients with DM and TB coexistence, accurately distinguishing between LTBI and active TB. This contributes to early screening of high‐risk patients, timely intervention, and prevention of disease progression. Moreover, machine learning can provide effective treatment plans and high‐quality prognosis assessments for patients by analyzing clinical information data and medical image data.

### 5.5 Clinical trials needed to validate DM‐LTBI/DM‐TB biomarkers

Some biomarkers associated with DM‐TB have been identified, but most have not been clinically validated. For example, Qi Yu et al. found that the monocyte‐to‐high‐density lipoprotein cholesterol ratio (MHR), neutrophil‐to‐high‐density lipoprotein cholesterol ratio (NHR), C‐reactive protein‐to‐lymphocyte ratio (CLR), and C‐reactive protein‐to‐albumin ratio (CAR) may serve as novel inflammatory biomarkers for predicting and evaluating active PTB in T2DM patients.^[^ ^222^ ^]^ Zhang et al. found a significant increase in NKT cells in the peripheral blood and bronchoalveolar lavage fluid of DM‐TB patients, which may indicate a convenient and noninvasive biomarker for DM‐TB.^[^ ^223^ ^]^ These candidate biomarkers selected based on omics or other methods for predicting, prognosticating, and evaluating treatment responses in DM‐TB have not yet undergone clinical trials. Therefore, more accurate, convenient, and efficient biomarkers need to be selected based on clinical validation to assist in clinical diagnosis and treatment.

## 6 CONCLUSIONS

In conclusion, this review highlights the significant burden posed by the coexistence of DM and TB on a global scale. Extensive research has demonstrated that DM is a major risk factor for TB. From an immunological perspective, it is evident that high blood glucose levels in TB patients contribute to decreased innate immune cells, impaired phagocytic function, and delayed antigen presentation. This, in turn, delays the initiation of adaptive immune responses and compromises the body's ability to clear MTB. Additionally, the interaction between TB and DM leads to heightened inflammation and elevated pro‐inflammatory cytokine levels, exacerbating the inflammatory response and resulting in increased pathological damage, ultimately impacting TB treatment outcomes.

Furthermore, the impact of DM on TB goes beyond pathogenesis. Poor glycemic control, IR, DM complications, and genetic factors all contribute to an increased risk of MTB infection. In addition, DM‐related immune suppression affects the sensitivity of traditional diagnostic tests for TB, potentially leading to underdiagnosis and missed opportunities for early intervention. To address the challenges posed by the co‐occurrence of DM and TB, further research is required to elucidate the underlying mechanisms of DM reactivation in LTBI. Early screening and intervention for LTBI in DM patients are crucial for reducing the risk of TB. Exploring specific biomarkers for DM‐LTBI can improve diagnostic accuracy, but these biomarkers need to be validated through rigorous clinical trials before implementation. Additionally, integrating machine learning techniques into clinical practice can offer valuable support in enhancing diagnostic and therapeutic strategies.

In summary, unraveling the complexities of DM‐TB comorbidity requires a multidisciplinary approach combining immunology, epidemiology, and clinical research. By deepening our understanding of the interplay between DM and TB, we can develop more effective preventive measures, accurate diagnostic tools, and targeted therapeutic interventions to alleviate the global burden imposed by these two interconnected diseases.

## AUTHOR CONTRIBUTIONS

Zhaoyang Ye: Data curation; methodology; software; writing—original draft. Linsheng Li: Data curation; methodology. Ling Yang: Data curation; methodology. Li Zhuang: Data curation; methodology. Ashok Aspatwar: Writing—review & editing. Liang Wang: Conceptualization; writing—review & editing. Wenping Gong: Conceptualization; funding acquisition; software; writing—review & editing.

## CONFLICT OF INTEREST STATEMENT

The authors declare no conflicts of interest. The funders had no role in the design of the study; in the collection, analyses, or interpretation of data; in the writing of the manuscript, or in the decision to publish the results.

## Data Availability

All data generated or analyzed during this study are included in this published article.
